# Transcriptome analysis revealed the genes and major pathways involved in prunetrin treated hepatocellular carcinoma cells

**DOI:** 10.3389/fphar.2024.1400186

**Published:** 2024-11-01

**Authors:** Abuyaseer Abusaliya, Hun Hwan Kim, Preethi Vetrivel, Pritam Bhagwan Bhosale, Se Hyo Jeong, Min Yeong Park, Si Joon Lee, Gon Sup Kim

**Affiliations:** ^1^ Department of Veterinary Medicine, Research Institute of Life Science, Gyeongsang National University, Jinju, Republic of Korea; ^2^ Preclinical Research Center, Daegu-Gyeongbuk Medical Innovation Foundation, Daegu, Republic of Korea

**Keywords:** hepatocellular carcinoma, Hep3B cells, transcriptome sequencing, RNA-seq, prunetrin

## Abstract

Liver cancer represents a complex and severe ailment that poses tough challenges to global healthcare. Transcriptome sequencing plays a crucial role in enhancing our understanding of cancer biology and accelerating the development of more effective methods for cancer diagnosis and treatment. In the course of our current investigation, we identified a total of 1,149 differentially expressed genes (DEGs), encompassing 499 upregulated and 650 downregulated genes, subsequent to prunetrin (PUR) treatment. Our methodology encompassed gene and pathway enrichment analysis, functional annotation, KEGG pathway assessments, and protein-protein interaction (PPI) analysis of the DEGs. The preeminent genes within the DEGs were found to be associated with apoptotic processes, cell cycle regulation, the PI3k/Akt pathway, the MAPK pathway, and the mTOR pathway. Furthermore, key apoptotic-related genes exhibited close interconnections and cluster analysis found three interacting hub genes namely, *TP53*, *TGFB1* and *CASP8*. Validation of these genes was achieved through GEPIA and western blotting. Collectively, our findings provide insights into the functional landscape of liver cancer-related genes, shedding light on the molecular mechanisms driving disease progression and highlighting potential targets for therapeutic intervention.

## 1 Introduction

Liver cancer is a global health problem that is rapidly increasing in prevalence, particularly in Western countries. Chemotherapeutic agents are essential in the treatment of various types of cancer, but they often come with a range of side effects (Carr et al., 2008; Nurgali et al., 2018; Zhao et al., 2022). These side effects can significantly impact the quality of life of cancer patients undergoing chemotherapy (Mohammedi et al., 2008). It is important for healthcare providers to closely monitor and manage these side effects to ensure the best possible outcomes for patients. Additionally, some chemotherapeutics can also cause long-term side effects such as organ damage, fertility issues, and an increased risk of developing secondary cancers (Brianna and Lee, 2023; Phillips et al., 2023). The diagnostic challenges associated with liver cancer further compound the issue. Liver cancer presents a diagnostic challenge due to its diverse etiology and lack of specific symptoms in the early stages (Bakrania et al., 2023; Omar et al., 2023). Moreover, differentiating primary liver cancer from secondary liver cancer can be challenging due to overlapping imaging features, similar histopathological characteristics, and the lack of specific biomarkers, metastatic lesions can mimic primary liver tumors in appearance, and molecular profiles may not clearly differentiate between the two. These factors complicate accurate diagnosis and require a comprehensive approach integrating clinical, radiological, and molecular data (Zhou et al., 2023).

Transcriptome sequencing, also known as RNA-Seq, is a powerful tool used to analyze and quantify the complete set of RNA molecules present in a sample (Deshpande et al., 2023). This technique allows researchers to study gene expression patterns and identify differentially expressed genes in various biological conditions, including cancer (Hong et al., 2020). The availability of transcriptome sequencing data has greatly contributed to the understanding of cancer biology and has led to the discovery of novel biomarkers and therapeutic targets (Pedersen and Kanigan, 2016; Wang et al., 2020). Furthermore, transcriptome sequencing has revolutionized cancer research by providing insights into the intricate mechanisms underlying tumor development, progression, and response to treatment (Guan et al., 2012; Fumagalli et al., 2017).

By analysing the transcriptome, researchers can identify specific genes and pathways that are dysregulated in cancer cells, providing valuable information for the development of targeted therapies and personalized treatment approaches (Ho et al., 2015; Kori and Yalcin Arga, 2018). By comparing the transcriptome profiles of cancer cells to normal cells, researchers can also identify potential diagnostic markers that can aid in early detection and prognosis of cancer (Larson et al., 2021; Yasui et al., 2009). The continual advancements in transcriptome sequencing technologies, data analysis methods, and integrative multi-omics approaches hold promise for further uncovering the intricate molecular networks governing cancer pathogenesis and for translating these findings into clinical applications (Moncada et al., 2020; Satam et al., 2023; Tsakiroglou et al., 2023).

**FIGURE 1:**
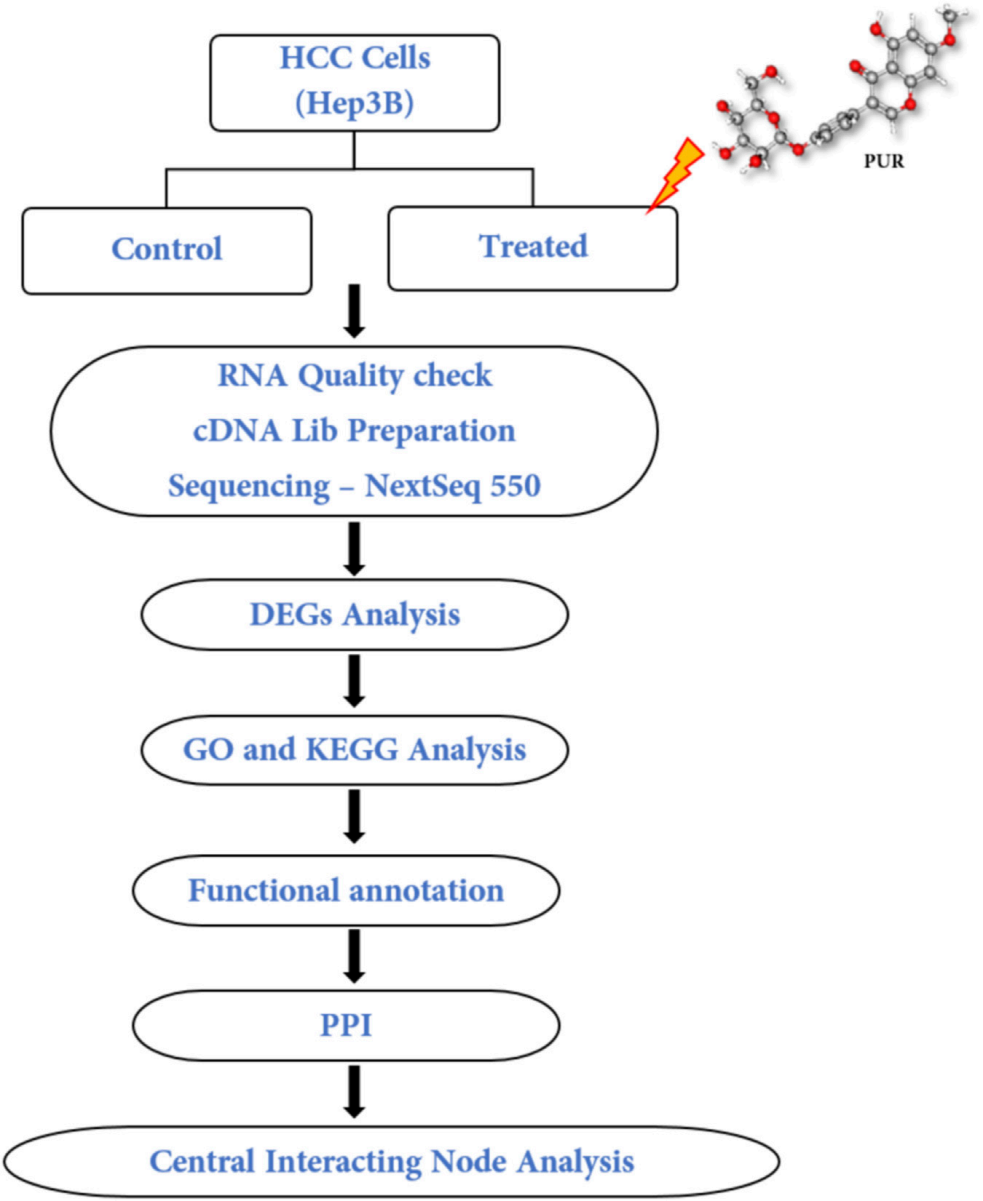
Schematic illustration of the stepwise workflow adopted in current study.

Prunetrin (Prunetin 4′-O-glucoside; PUR) is a glycosyloxyisoflavone. Its precursor, prunetin, has been demonstrated to induce necroptotic cell death in gastric cancer, as evidenced by next-generation sequencing (Vetrivel et al., 2022). Additionally, prunetin in its glycosidic form, known as prunetinoside, has shown therapeutic effects by targeting gastric cancer cells (Vetrivel et al., 2021). Furthermore, our previous study has revealed PUR’s ability to induce apoptosis in Hep3B cells (Abusaliya et al., 2023). Hence, the primary objective of the present investigation is to delineate the gene expression profile in Hep3B hepatocellular carcinoma (HCC) cells subjected to PUR treatment. Employing a comprehensive sequencing approach, the Illumina NextSeq550 platform was utilized to discern differentially expressed genes within the comparative contexts of control and PUR-treated conditions. The comprehensive schematic illustration of the stepwise workflow employed in the study, offering a visual representation of the experimental design and analytical processes, is encapsulated in Figure 1.

## 2 Results

### 2.1 Cell viability and morphological characteristics associated with PUR treatment

**FIGURE 2:**
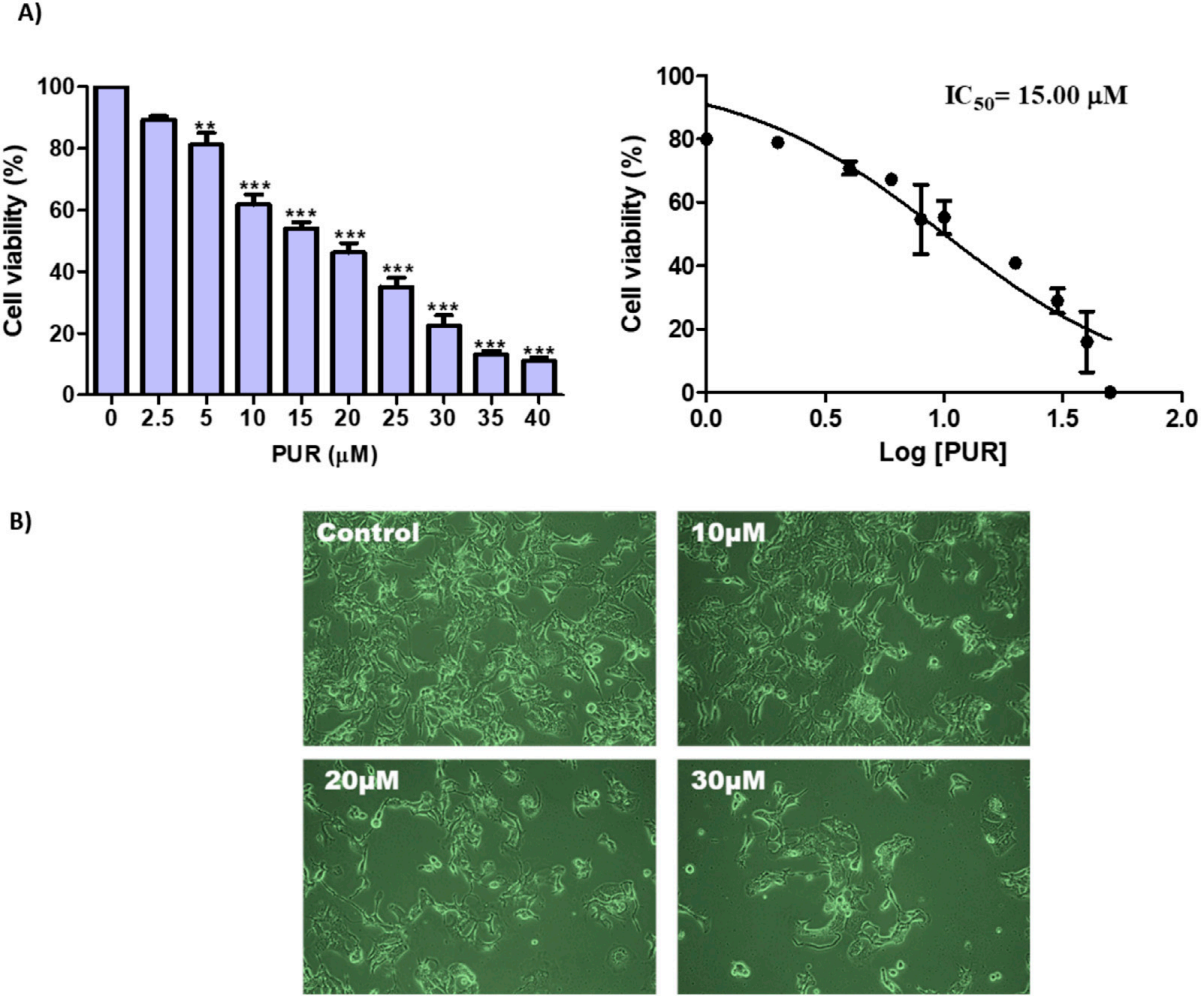
Effect of PUR on cell viability. **(A)** Cytotoxicity Assessment in Hep3B Cells; **(B)** Morphological Alterations Induced by Prunetrin Treatment. Cell viability was evaluated using MTT assays following a 24-hour exposure to varying concentrations of prunetrin (0, 2.5, 5, 10, 15, 20, 25, 30, 35, and 40 μM). For morphological analysis, cells were treated with prunetrin at concentrations of 10, 20, and 30 μM for 24 h, after which they were examined under an inverted microscope. The results are expressed as mean ± standard error of the mean (SEM). Statistical significance was denoted as follows: ** for *p* < 0.01, and *** for *p* < 0.001.

To assess the cytotoxic potential of PUR in Hep3B cells, MTT assay was conducted. The results depicted in Figure 2A demonstrate that PUR induces cytotoxic effects in cancer cells, leading to significant dose-dependent inhibition of cell proliferation, with an observed IC50 value of 15.0 μM for 24 h. Additionally, morphological alterations were examined under a light microscope. As illustrated in Figure 2B, cells treated with PUR exhibited characteristic features of cell death, such as cellular shrinkage, irregular shape, and cytoplasmic blebbing, in comparison to untreated control cells. Moreover, a dose-dependent decrease in cell counts was observed in PUR-treated cells, with a notable increase in the number of detached cells.

### 2.2 Differentially expressed genes (DEGs) analysis

**FIGURE 3:**
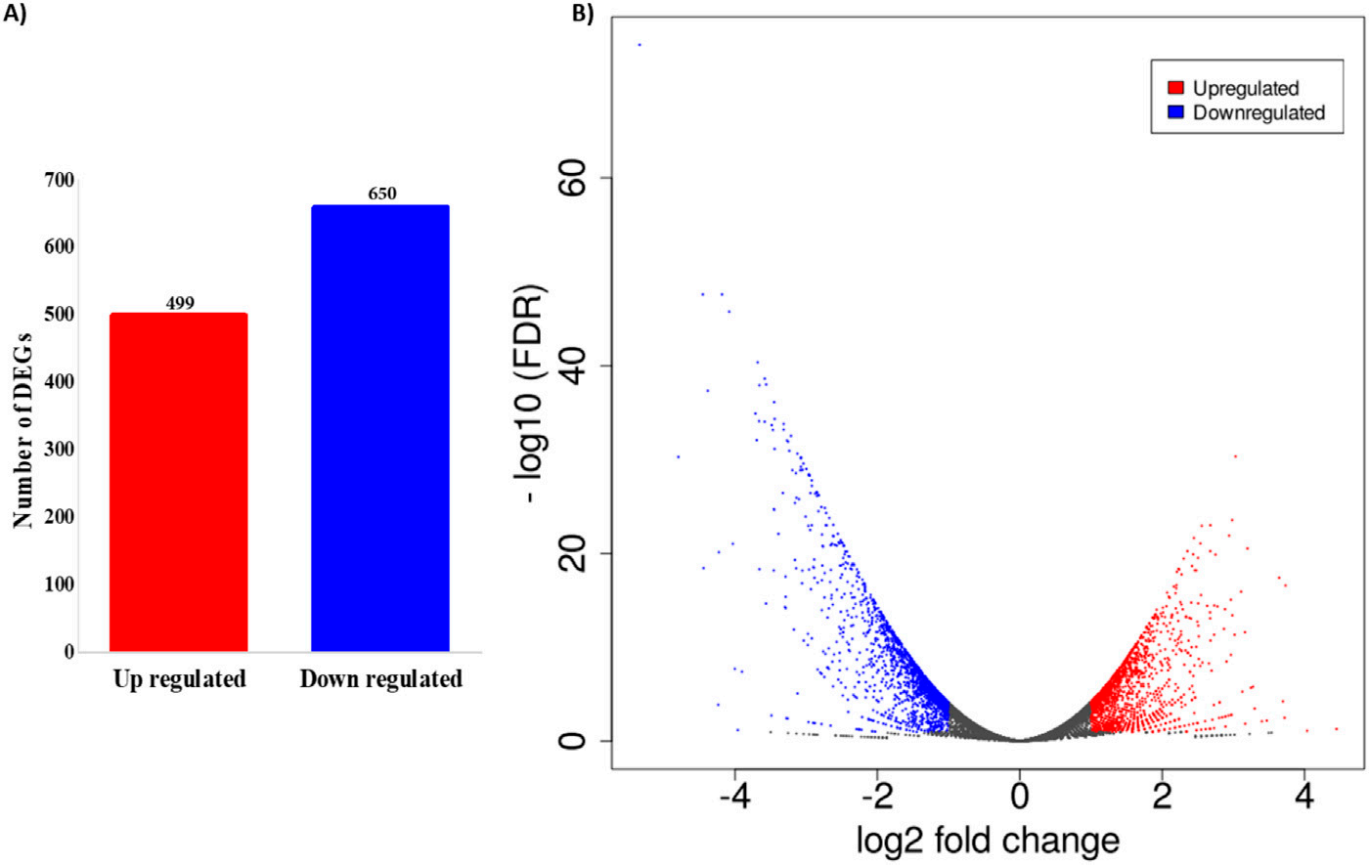
Differentially expressed genes (DEGs) analysis. **(A)** Number of DEGs among control and treated. A total of 499 up-regulated and 650 down-regulated differentially expressed genes was found between PUR-treated and control samples; **(B)** Scatter plot analysis displaying the distinct pattern of the genes between PUR-treated and control samples.

The total read mapping statistics of all the samples are represented in Supplementary Table S1. The DEGs elicited by PUR treatment in comparison to control cells underwent analysis by ExDEGA (v.5.0.0) (Ebiogen, Korea). Genes exhibiting a log_2_-fold-change ≥ 1 and a false discovery rate (FDR) below 0.05 in pairwise comparison between two conditions were identified and selected as DEGs. These criteria were chosen to balance the identification of biologically meaningful changes in gene expression with statistical rigor. Consequently, a total of 1,149 genes exhibited significant differential expression between the control and treated cellular conditions. Among these, 499 genes demonstrated an up-regulated expression pattern, while 650 genes displayed down-regulated expression, as shown in Figure 3A. Employing a volcano plot analysis based on logFC and false discovery rate (FDR < 0.05) values, Figure 3B illustrates the distinct expressions of each gene. In this representation, the blue colour signifies higher gene expression, whereas the red colour signifies lower gene expression, providing a comprehensive overview of the differential expression landscape between control and treated cells.

### 2.3 Gene ontology analysis of DEGs

**FIGURE 4:**
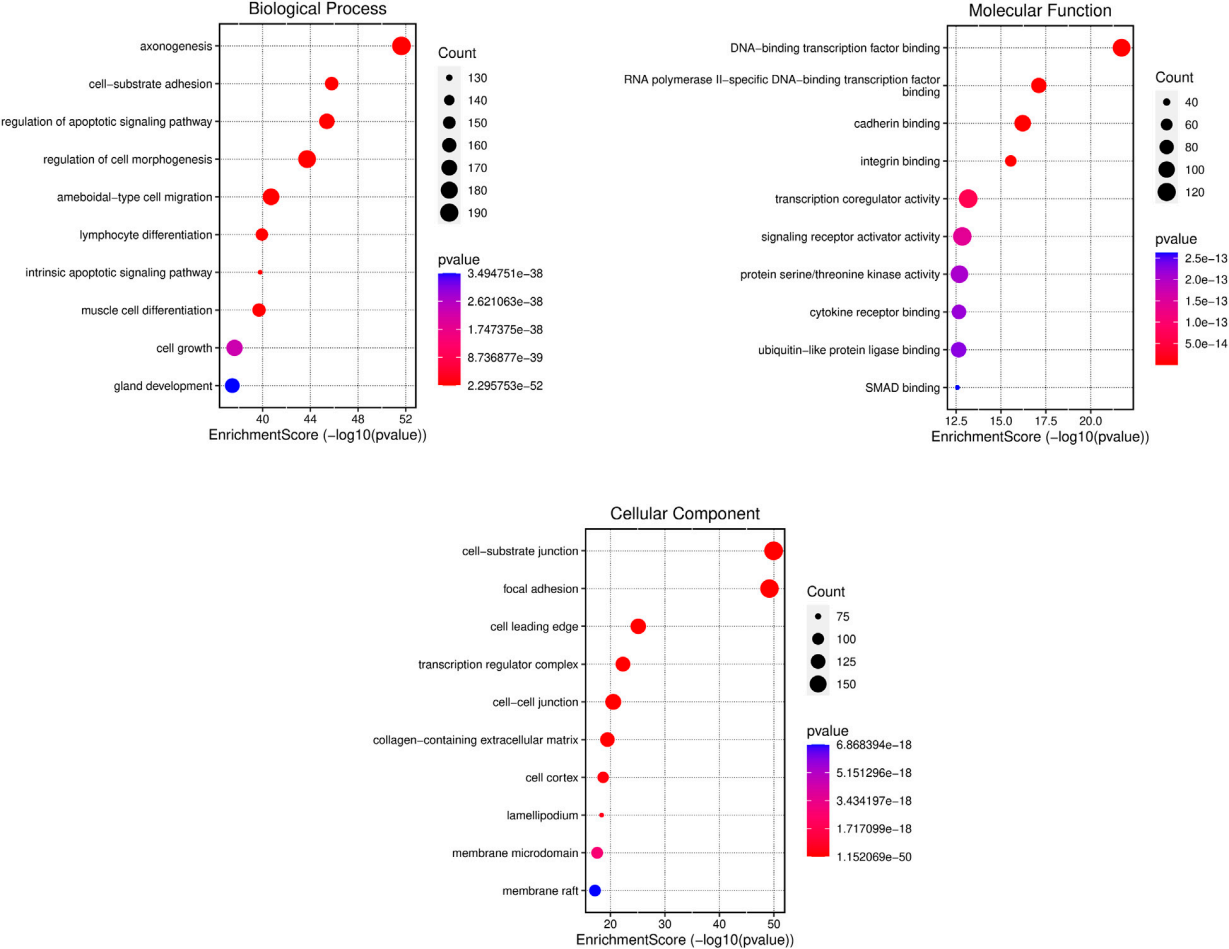
Gene ontology analysis of differentially expressed genes (DEGs). The *x-axis* is the enrichment score and *y-axis* shows the name of the process or function. The colour and size of the dots were based on the *p*-value and number of gene count.

Metascape was employed for Gene Ontology (GO) analysis of DEGs across three distinct categories: biological process, cellular components, and molecular function. The resultant findings, illustrated in Figure 4, present the top 10 GO terms within each category. In terms of biological processes, the DEGs demonstrated enrichment in cell-substrate adhesion, regulation of the apoptotic signaling pathway, regulation of cell morphogenesis, and intrinsic apoptotic signaling pathway. Similarly, within cellular components, enrichment was observed in cell-substrate junctions, focal adhesions, cell-cell junctions, and membrane rafts. In the domain of molecular function, the DEGs exhibited enrichment in DNA-binding transcription factor binding, cadherin binding, integrin binding, and transcription coregulatory activity. This comprehensive analysis provides insights into the functional roles and interactions of the identified DEGs across various biological contexts.

### 2.4 GO enrichment analysis between up-regulated and down-regulated genes

**FIGURE 5:**
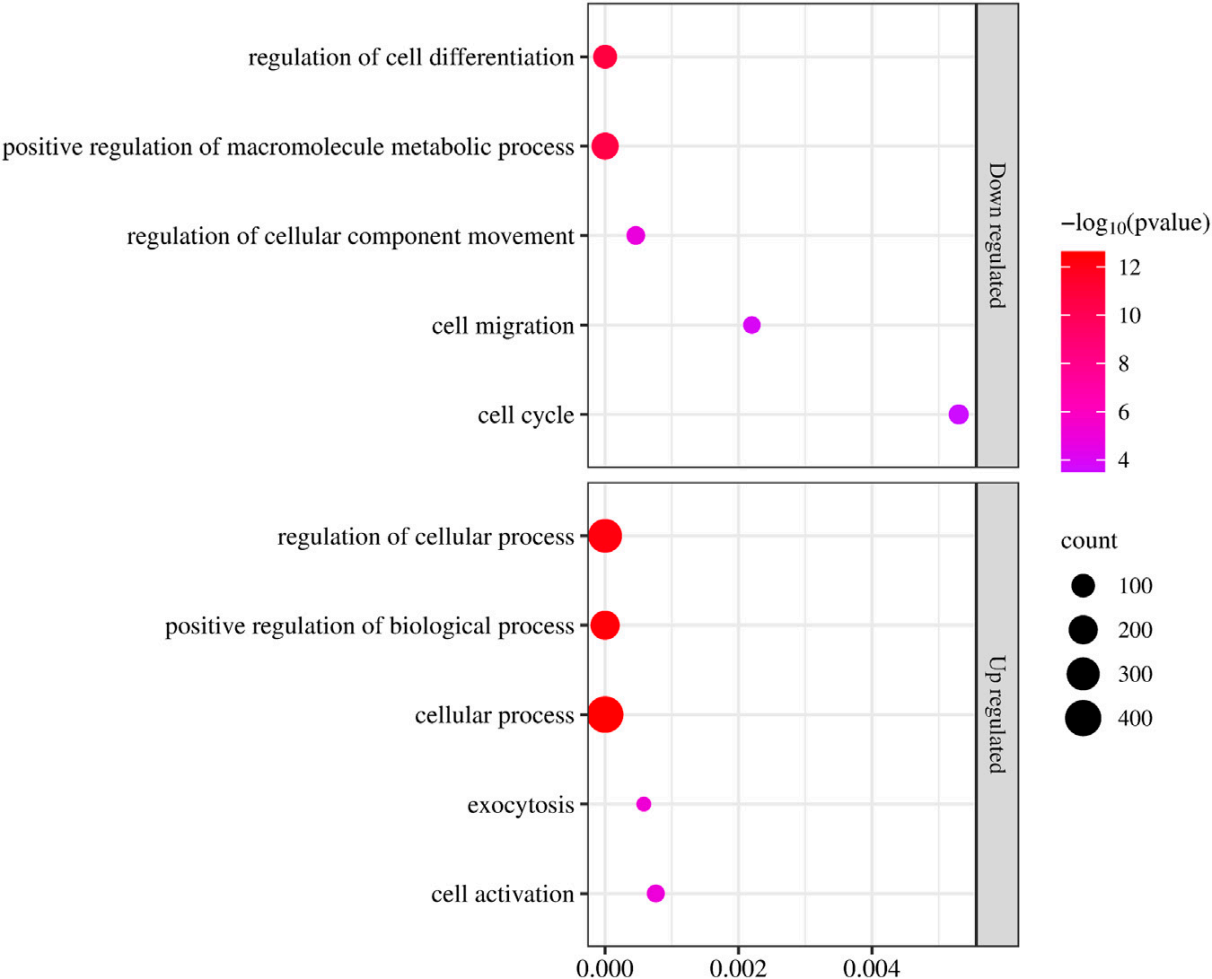
Gene ontology enrichment analysis of DEGs displaying upregulated and downregulated DEGs with–log_10_ and number of counts. The *x-axis* shows the *p*-value and *y-axis* shows the name of the process or function. The colour and size of the dots were based on the *p*-value and number of genes count. Red represents high significance, while pink represents low.

Further, GO enrichment analysis were systematically conducted across three distinct categories. The culmination of these analysis was represented in a bubble plot, integrating *p* values for both upregulated and downregulated genes (Figure 5). The findings unveiled a downregulation in processes associated with the regulation of cell differentiation, positive regulation of macromolecule metabolic processes, cell migration, and cell cycle. Conversely, an upregulation was observed in the GO terms related to the regulation of cellular processes, positive regulation of biological processes, exocytosis, and cell activation. These findings collectively suggest a comprehensive restructuring of cellular functions in response to the PUR treatment. This underscores the intricate regulatory mechanisms of the dynamic nature of cellular responses and the potential implications for various physiological and pathological processes.

### 2.5 Functional annotation analysis

For comprehensive functional annotation, DEGs underwent analysis using GeneCodis, which provides a comprehensive overview of the functional attributes of the gene set, emphasizing significantly enriched biological processes, molecular functions and cellular components networks as illustrated in Supplementary Figure S1. These findings facilitate the interpretation of the gene set and offer insights into the potential roles of these genes in response to PUR treatment. The top 10 Gene Ontology (GO) terms characterized by low p-values and closely associated networks were delineated in Tables 1–3. In the biological process category, annotated terms included apoptotic process (GO:0006915), positive regulation of apoptotic process (GO:0043065), apoptotic mitochondrial changes (GO:0008637), and positive regulation of release of cytochrome c from mitochondria (GO:0090200). Similarly, in the cellular components category, highlighted terms encompassed Bcl-2 family protein complex (GO:0097136), mitochondrion (GO:0005739), cytoplasm (GO:0005737), and cytosol (GO:0005829). Molecular function annotations revealed terms such as death receptor binding (GO:0005123), identical protein binding (GO:0042802), type III transforming growth factor beta receptor binding (GO:0034714), BH3 domain binding (GO:0051434), and protein kinase binding (GO:0019901) was found. These network predictions offer insights into the functional roles and interactions of DEGs across various biological contexts, emphasizing the significance of apoptotic processes in the observed gene expression alterations.

**TABLE 1:** Top 10 significant GO terms of DEGs in terms of biological process.

Description	GO term	P-Value	Genes
Apoptotic process	GO:0006915	2.09E-10	*DAB2IP, TNFSF10, BCL2L1, BID, TGFB1, BAX, TGFBR2, PYCARD, CDKN2A, FADD, FHIT*
Positive regulation of apoptotic process	GO:0043065	4.01E-09	*DAB2IP, TNFSF10, TP53, BCL2L1, BID, BAX, PYCARD, CDKN2A, FADD*
Apoptotic mitochondrial changes	GO:0008637	6.12E-09	*BCL2L1, BID, BAX, CDKN2A*
Positive regulation of protein-containing complex assembly	GO:0031334	6.49E-09	*DAB2IP, BID, TGFB1, BAX, VEGFA*
Positive regulation of protein phosphorylation	GO:0001934	7.44E-09	*IGF2, PTK2, RELN, TGFB1, RAC1, VEGFA, CCND1*
Positive regulation of release of cytochrome c from mitochondria	GO:0090200	3.48E-08	*TNFSF10, BID, BAX, PYCARD*
Positive regulation of extrinsic apoptotic signaling pathway	GO:2001238	4.71E-08	*TNFSF10, BID, PYCARD, FADD*
Negative regulation of fibroblast proliferation	GO:0048147	5.43E-08	*MYC, DAB2IP, GSTP1, BAX*
Release of cytochrome c from mitochondria	GO:0001836	2.39E-06	*BCL2L1, BID, BAX*
Positive regulation of phosphatidylinositol 3-kinase signaling	GO:0014068	3.15E-06	*PTK2, RELN, EGF, VEGFA*

**TABLE 2:** Top 10 significant GO terms of DEGs in terms of cellular components.

Description	GO term	P-Value	Genes
Bcl-2 family protein complex	GO:0097136	3.96E-05	*BCL2L1, BAX*
Mitochondrion	GO:0005739	5.23E-05	*MYC, GSTP1, TERT, BCL2L1, BID, BAX, PYCARD, CDKN2A, FHIT*
Cytoplasm	GO:0005737	7.16E-05	*MYC, DAB2IP, GSTP1, TERT, PTK2, RELN, BCL2L1, BID, TGFB1, BAX, TGFBR2, PYCARD, RAC1, CDKN2A, FADD, VEGFA, FHIT, CCND1*
Extracellular region	GO:0005576	0.000275,519	*GSTP1, MET, IGF2, TNFSF10, RELN, TGFB1, EGF, PYCARD, VEGFA, ANGPT2*
Extracellular space	GO:0005615	0.000898,234	*GSTP1, IGF2, TNFSF10, RELN, TGFB1, EGF, VEGFA, ANGPT2*
Cytosol	GO:0005829	0.001,061,733	*DAB2IP, GSTP1, TERT, PTK2, BCL2L1, BID, BAX, TGFBR2, PYCARD, RAC1, CDKN2A, FADD, FHIT, CCND1*
Mitochondrial membrane	GO:0031966	0.001,189,134	*BCL2L1, BID, BAX*
BAX complex	GO:0097144	0.001218027	*BAX*
Mitochondrial outer membrane	GO:0005741	0.001673487	*BCL2L1, BID, BAX*
BAK complex	GO:0097145	0.002434634	*BAX*

**TABLE 3:** Top 10 significant GO terms of DEGs in terms of molecular function.

Description	GO term	P-value	Genes
Death receptor binding	GO:0005123	1.19244E-06	*DAB2IP, BID, FADD*
Identical protein binding	GO:0042802	1.96582E-06	*DAB2IP, MET, TERT, TNFSF10, BCL2L1, TGFB1, BAX, PYCARD, FADD, VEGFA, FHIT*
Type III transforming growth factor beta receptor binding	GO:0034714	9.19027E-06	*TGFB1, TGFBR2*
BH3 domain binding	GO:0051434	2.29403E-05	*BCL2L1, BAX*
Protein kinase binding	GO:0019901	2.30089E-05	*DAB2IP, PTK2, BCL2L1, RAC1, CDKN2A, CCND1*
Tumor necrosis factor receptor superfamily binding	GO:0032813	3.20916E-05	*TNFSF10, FADD*
Growth factor activity	GO:0008083	4.73177E-05	*IGF2, TGFB1, EGF, VEGFA*
JUN kinase binding	GO:0008432	5.49295E-05	*GSTP1, PTK2*
Vascular endothelial growth factor receptor 2 binding	GO:0043184	5.49295E-05	*DAB2IP, VEGFA*
Protein-containing complex binding	GO:0044877	6.79995E-05	*MYC, DAB2IP, RAC1, FADD, CCND1*

### 2.6 KEGG pathway enrichment analysis of DEGs

**FIGURE 6:**
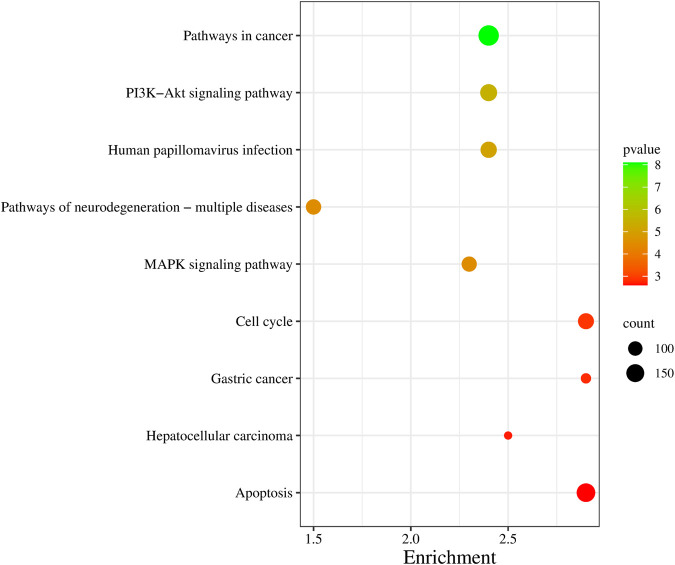
Enrichment of KEGG pathway based on the count and *p*-value. The *x-axis* shows the enrichment score and *y-axis* shows the name of the pathway. The colour and size of the dots were based on the *p*-value and number of genes count. Green represents high significance, while red represents low.

To elucidate the pathways implicated in the DEGs, a pathway enrichment analysis was conducted utilizing the Kyoto Encyclopaedia of Genes and Genomes (KEGG). The results, showcased in Figure 6, present the top 10 enriched pathways. Notably, the top significantly enriched pathways, as detailed in Supplementary Table S2, exhibited based on *P* values and the number of gene counts. These pathways encompassed crucial biological processes, including but not limited to pathways in cancer, PI3K-Akt signaling pathway, MAPK signaling pathway, cell cycle, and apoptosis. Alterations in the PI3K-Akt signaling pathway could shed light on mechanisms of tumor growth or resistance, while changes in the MAPK signaling pathway might offer insights into cellular responses to stress or proliferation. Additionally, understanding shifts in cell cycle and apoptosis pathways can reveal how gene expression changes affect cellular turnover and survival. The comprehensive identification of these pathways found that the molecular mechanisms underlying the observed gene expression alterations, provide valuable insights into the potential functional roles of the DEGs in the context of these pathways.

### 2.7 Apoptotic gene expression analysis by KEGG

**FIGURE 7:**
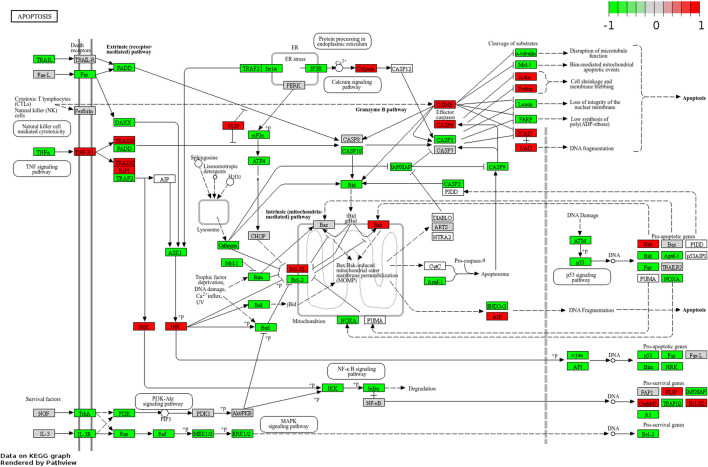
KEGG pathway mapping of genes involved in apoptosis pathway based on the *p*-value and dark red colour denotes high expression while green denotes low expression.

Following the preceding analysis, which identified apoptotic-related genes and the apoptotic pathway as significantly prominent, a detailed exploration of the expression patterns of apoptotic-related genes was conducted. Utilizing the KEGG, the apoptotic pathway was mapped, and the expression patterns of relevant genes were visualized through the Pathview R tool. The result shown in Figure 7, highlights a predominance of upregulated expression among the genes participating in the apoptotic pathway. This observation underscores the potential activation or modulation of apoptotic processes and the regulatory dynamics of apoptosis-related genes and their potential significance in PUR treated cells.

### 2.8 Protein-protein interaction and module network construction

**FIGURE 8:**
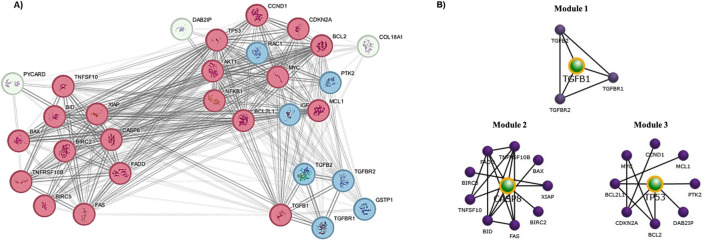
Protein-Protein Interaction (PPI) analysis and Module network construction. **(A)** Clustering analysis using the STRING-Cytoscape by MCODE plugin (Red-high interaction; Blue- Moderate; White-low); **(B)** Central target proteins prediction by FunRich module network construction.

The Protein-Protein Interaction (PPI) analysis conducted using the STRING database from genes selected based on functional annotation revealed a network comprising 30 nodes and 230 edges, with a high clustering coefficient value of 0.799 and a significant enrichment p-value of < 1.0e-16 (Supplementary Figure S2) and the identified network was visualized using Cytoscape. Further, clustering analysis using the MCODE plugin in Cytoscape was employed to identify densely interconnected clusters (Figure 8A). Subsequently, module network construction was performed using FunRich, a functional enrichment analysis tool to determine the core hub targets of each cluster. This clearly revealed three specific targets *TP53*, *TGFB1*, and *CASP8* within the network with the maximum interaction between other proteins (Figure 8B).

### 2.9 Assessment of mRNA and protein expression of target genes

**FIGURE 9:**
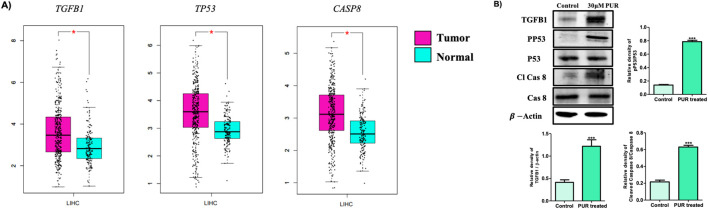
Assessment of mRNA and protein expression of target genes. **(A)** mRNA analysis via TCGA (The Cancer Genome Atlas-Liver Hepatocellular Carcinoma) and GTEx by GEPIA; **(B)** For protein expression, following a 24-hour exposure to 30 μM PUR, cellular proteins were isolated by SDS-PAGE (10% gel were used for all proteins) for subsequent Western blotting analysis. Densitometry was employed to quantify expression levels, utilizing the standard error of the mean (SEM) calculated from three independent values. Protein expression was normalized using β-actin. The outcomes are depicted as the mean ± SEM, with significance levels denoted as ***p < 0.001.

The mRNA expression levels of all the genes from the three network clusters were systematically investigated through the utilization of the TCGA (The Cancer Genome Atlas-Liver Hepatocellular Carcinoma) and GTEx by GEPIA (Supplementary Figure S3). Focusing on identified pivotal targets, namely *TP53*, *TGFB1*, and *CASP8*, the analysis was performed employing the LIHC (Liver Hepatocellular Carcinoma) as a reference dataset. The outcome, as illustrated in Figure 9A, distinctly showcased a substantial upregulation in mRNA expression levels within LIHC tissues in comparison to their normal tissues. The results derived from the GEPIA database underscored a statistically significant elevation (*p* < 0.01) in mRNA expression levels for all three targets in LIHC tissues relative to normal tissues. However, our western blotting analysis reveals significant activation and increased expression levels of all three proteins subsequent to treatment with PUR. This substantiates the activation of these central target proteins by PUR (Figure 9B). Thus, these findings suggest that PUR has the capacity to induce apoptosis in Hep3B cancer cells via the identified hub targets. This evidence underscores the potential therapeutic utility of PUR in modulating crucial signaling pathways associated with apoptosis induction in Hep3B cancer cells.

## 3 Discussion

Transcriptome sequencing plays a pivotal role in liver cancer research by providing comprehensive insights into the dynamic gene expression patterns underlying the development and progression of the disease (Govaere et al., 2020; Wu et al., 2021). Through transcriptome analysis, researchers can identify dysregulated genes, alternative splicing events, and non-coding RNAs associated with hepatocarcinogenesis, thus elucidating key molecular pathways and potential therapeutic targets (Kori and Yalcin Arga, 2018; Li S. et al., 2019). Additionally, it enables the stratification of liver cancer subtypes based on gene expression signatures, facilitating personalized treatment strategies and prognostic assessments. Furthermore, transcriptome sequencing facilitates the exploration of tumor microenvironment interactions, immune evasion mechanisms, and drug resistance mechanisms in liver cancer, ultimately contributing to the advancement of precision medicine approaches and the development of novel diagnostic and therapeutic interventions for this heterogeneous malignancy (Cummings et al., 2017; Li W. et al., 2019; Natua et al., 2022). Transcriptome sequencing has been instrumental in the identification of alternative splicing events, non-coding RNAs, and fusion genes, shedding light on the complex molecular landscape of cancer (Maher et al., 2009; Mutz et al., 2013; Casamassimi et al., 2017). In our previously reported study, we elucidated the pharmacological action of PUR by presenting *in vitro* evidence of its capability to induce apoptotic cell death in Hep3B cells (Abusaliya et al., 2023). For further exploration, we adopted NGS analysis to investigate deeper into the molecular mechanism of PUR induced apoptotic cell death. The primary objective of the study is to gain comprehensive insights into the differential gene expression profile elicited by PUR treatment, with a specific focus on identifying potential candidate biomarkers.

The transcriptomic impact on cellular gene expression was systematically investigated. As a result, a comprehensive analysis revealed a total of 1,149 genes displaying statistically significant differential expression in response to PUR treatment when contrasted with control cells. Specifically, the upregulation of 499 genes indicates the activation of cellular processes related to cell death, stress responses, or metabolic changes. Conversely, the downregulation of 650 genes suggests the suppression of pathways crucial for growth, survival, or treatment response, which might be detrimental under experimental conditions. These findings are directly aligned with the goal of the study to identify potential targets for therapeutic intervention, biomarkers for diagnosis, or key regulators of disease progression.

The top 10 enriched GO terms, indicative of significant functional annotations, were selected for each category (biological processes, molecular function, and cellular components). These terms collectively provide insights into the diverse roles and associations of the identified liver cancer-related genes within cellular and molecular contexts. Our results show that in terms of biological processes, the differentially expressed genes (DEGs) displayed enrichment in several key functional categories. Enrichment was observed in processes related to cell-substrate adhesion, which underscores the importance of interactions between cells and their extracellular environment. The regulation of the apoptotic signaling pathway, cell morphogenesis, and intrinsic apoptotic signaling pathway emerged as enriched biological processes, suggesting a dynamic interplay between cell survival, morphology, and regulatory mechanisms and highlighting the involvement of intrinsic cellular mechanisms in regulating programmed cell death.

Within cellular components, the DEGs demonstrated enrichment in structures crucial for cell adhesion and communication. Specifically, enrichment was observed in cell-substrate junctions, focal adhesions, membrane rafts and cell-cell junctions, emphasizing the significance of these structures in maintaining tissue integrity, facilitating cellular interactions and the potential involvement of specialized membrane microdomains in cellular processes associated with liver cancer. In the domain of molecular function, the DEGs exhibited enrichment in molecular interactions critical for gene regulation and cell adhesion. DNA-binding transcription factor binding indicates the involvement of transcriptional regulation in modulating gene expression patterns associated with liver cancer. Similarly, enrichment in cadherin binding, integrin binding and transcription coregulatory activity highlights the importance of cell adhesion molecules in mediating cell-cell, cell-extracellular matrix interactions and involvement of regulatory proteins in modulating transcriptional responses underlying liver cancer pathogenesis.

To enhance the interpretability of these findings, a gene functional annotation cluster network was meticulously constructed, visually encapsulating the relationships and interactions among the enriched terms across biological processes, molecular functions, and cellular components. The network predictions derived from these analysis collectively offer comprehensive insights into the functional roles and interactions of DEGs across various biological contexts. Notably, the emphasis on apoptotic processes in the identified gene expression alterations suggests a significant role of apoptosis-related pathways in the observed molecular changes.

Our investigation uncovered substantial changes in cellular processes, indicating a nuanced response to the experimental conditions. Firstly, we observed a noteworthy downregulation in pathways related to the regulation of cell differentiation. This suggests a potential impairment in the cellular mechanisms governing the specialization of cells into distinct lineages, which could have implications for tissue homeostasis and development. We found there was a decrease in the positive regulation of macromolecule metabolic processes. This alteration hints at a potential slowdown in the biosynthesis and turnover of essential cellular components, which could impact various physiological functions such as energy production, signal transduction, and cellular repair processes. Moreover, we observed a decrease in pathways associated with cell migration. This finding suggests a potential attenuation in the ability of cells to move within tissues, which could affect the process of cancer metastasis and there was a downregulation observed in the cell cycle process, indicating a potential deceleration in the progression of cells through the various phases of the cell cycle. This alteration could have implications for cell proliferation, differentiation, and genomic stability under the PUR treatment.

Conversely, we noted an upregulation in gene ontology terms related to the regulation of various cellular processes. This suggests an increased emphasis on the control and coordination of cellular activities in response to the experimental conditions, possibly reflecting a compensatory mechanism to maintain cellular homeostasis. Furthermore, there was an increase in the positive modulation of biological activities, implying an enhanced activation of cellular functions and pathways involved in sustaining cellular viability and function. An upregulation was observed in pathways related to exocytosis, indicating a potential increase in the secretion of cellular components and signaling molecules, which could play crucial roles in intercellular communication and tissue remodeling. Also, we observed an increase in pathways associated with cell activation mechanisms, suggesting a heightened responsiveness of cells to various stimuli and signals in the microenvironment upon PUR treatment.

The integration of PPI analysis and module network construction facilitates a comprehensive exploration of protein interactions and network organization, contributing to our understanding of the molecular mechanisms underlying complex biological processes and found that *TP53*, *TGFB1* and *CASP8* as the central nodes. These central nodes may serve as key regulators or mediators of biological pathways or processes, warranting further investigation into their roles and potential therapeutic implications. The mRNA expression and protein expression observation emphasises the potential relevance of *TP53*, *TGFB1*, and *CASP8* in the context of liver cancer, suggesting their possible involvement in the molecular mechanisms underlying the pathogenesis of this particular malignancy.

TGFB1 is a multifunctional cytokine, that exhibits dual origins in HCC as it can be produced by HCC cells or the surrounding tumor stroma (Brenner et al., 2013). Notably, its significant metabolism and clearance primarily occur within the liver. The detection of *TGFB1* in the early stages of HCC underscores its potential as an early biomarker for the disease (Hanahan and Weinberg, 2011). The intricate interplay between cancer cells and the tumor microenvironment, coupled with the liver’s central role in *TGFB1* clearance, highlights the dynamic nature of *TGFB1* in the hepatocellular cancer (Yamashita et al., 2008; Hoshida et al., 2009). Understanding these aspects contributes to the exploration of *TGFB1* as a diagnostic indicator, providing valuable insights into the early stages of HCC and paving the way for potential early intervention strategies (Giannelli et al., 2014).

In liver cancer, *TP53* plays a crucial role and identification of *TP53* mutations or abnormal expression as a diagnostic marker improves the accuracy of diagnoses by characterizing the genomic landscape of malignancies (Liu and Gelmann, 2002; Robles and Harris, 2010). Within the prognostic domain, *TP53* mutations are powerful predictors, associated with increased disease progression and worse clinical outcomes (Kortekaas et al., 2020; Raffone et al., 2020). Monitoring *TP53* status informs prognostic evaluations and directs the development of personalized treatment plans. *TP53* is a target that shows potential for therapeutic intervention since it is at the core of cellular responses to DNA damage (Essmann and Schulze-Osthoff, 2012; Sabapathy and Lane, 2018). Targeted therapeutic interventions in liver cancer can be achieved by strategies that aim to modulate or restore *TP53* function, which could lead to breakthroughs in treatment approaches for this complex malignancy (Farnebo et al., 2010).

*CASP8* is a multifaceted player in liver cancer. The expression levels and activity may offer insights into the status of apoptotic pathways, allowing for a nuanced characterization of the molecular profile in liver cancer (Liao et al., 2015). Furthermore, *CASP8* alterations or deficiencies emerge as potent prognostic indicators, correlating with heightened disease aggressiveness and diminished clinical outcomes in affected patients (Olsson and Zhivotovsky, 2011; Mandal et al., 2020). Leveraging its central role in apoptotic regulation, *CASP8* stands out as a promising therapeutic target, prompting the exploration of strategies to restore or enhance its activity for interventions that induce apoptosis in cancer cells (Stupack, 2013). Beyond this, *CASP8*’s status serves as a biomarker guiding therapeutic responses, enabling tailored treatment strategies for cancer with compromised *CASP8* function.

The findings derived from our western blotting analysis reveal a notable activation and significantly increased expression of all three proteins following treatment with PUR. This compelling evidence substantiates the assertion that PUR actively triggers the activation of these central target proteins. The activation of these specific proteins, as demonstrated by our results, provides valuable molecular insights into the mechanistic pathways influenced by PUR treatment, thereby supporting its potential as an inducer of apoptosis in the context of Hep3B cancer cells. Collectively, these observations contribute to a deeper understanding of the molecular events associated with PUR treatment, emphasizing its potential therapeutic relevance in the modulation of critical signaling pathways linked to apoptosis and regulated cell death. The observed activation of key proteins associated with apoptosis provides a strong rationale for advancing PUR into subsequent clinical trials, where its efficacy, safety profile, and therapeutic potential can be rigorously assessed for future therapeutic applications in cancer treatment.

## 4 Limitations and conclusion

This study represents an initial pilot investigation building on our previous work. The limitations of our present study stem from practical constraints regarding resource availability and experimental feasibility. These constraints influenced our decision to opt for a single sample analysis. However, it's crucial to recognize that utilizing a single sample in scientific research, especially in exploratory or preliminary studies, can still provide valuable insights. Numerous studies documented in the scientific literature have effectively employed single-sample analysis to derive hypothesis and generate preliminary data, which subsequently underwent further investigation and validation. Despite the inherent limitation, our study adds significant insights to the current pool of knowledge. It sheds light on pertinent areas for future research, suggesting the necessity for larger sample sizes or additional replicates to strengthen the robustness and generalizability of our findings. The findings highlight specific aspects of gene expression changes and their potential implications for PUR treatment, offering a foundational understanding that can guide further investigations. This initial analysis has illuminated several key areas for future research, underscoring the importance of expanding upon our preliminary results to gain a more comprehensive understanding of the underlying mechanisms. One critical area for further exploration involves increasing the sample size to enhance the robustness and reliability of the results. Larger sample sizes are essential for capturing a broader range of biological variability and for confirming that the observed patterns are not unique to the single sample used. By including more samples, future studies can improve the reproducibility of the findings, ensuring that the results are consistent across different individuals and conditions. This approach will help validate the initial observations and provide a more accurate representation of how PUR treatment affects gene expression on a larger scale. Additionally, incorporating multiple replicates within the same study can further strengthen the validity of the results.

Collectively, this study contributes to the understanding of the molecular events associated with PUR treatment, emphasizing its potential therapeutic relevance in the modulation of critical signaling pathways linked to apoptosis and regulated cell death. The observed activation of key proteins associated with apoptosis provides a strong rationale for advancing PUR into subsequent clinical trials, where its efficacy, safety profile, and therapeutic potential can be rigorously assessed for future therapeutic applications in cancer treatment. Future research should include additional preclinical studies in diverse animal models to validate findings and evaluate off-target effects, along with trials using varied dosing regimens to determine the optimal therapeutic window. Longitudinal studies are also recommended to evaluate long-term safety and efficacy. Exploring biomarkers for treatment response and testing combination therapies could further enhance outcomes.

## 5 Materials and methods

### 5.1 Cell culture maintenance and PUR treatment

The Hep3B cell line (ATCC Cat# HB-8064) was procured and propagated in Dulbecco’s Modified Eagle Medium (DMEM) augmented with fetal bovine serum (FBS) at a concentration of 10% (v/v), along with streptomycin (100 μg/mL) and penicillin (100 U/mL) obtained from Gibco (Thermo Fisher Scientific). The cellular cultures were maintained in a controlled environment set at 37°C, characterized by a humidified atmosphere containing 5% carbon dioxide (CO2). For the treatment with PUR, cells were initially seeded at a density of 4 × 10^5^ cells per culture vessel. Subsequently, PUR was added to the culture medium and the cells were incubated for 24 h according to standard protocols.

### 5.2 Total RNA isolation

To obtain the total RNA, the cells were washed with 1 × PBS after 24 h of treatment. Total RNA was isolated using Trizol reagent (Invitrogen) from the two samples (treated and control cells) and isolated RNA was dissolved in Diethylpyrocarbonate-treated water (iNtRON Biotechnology). The Agilent TapeStation 4,000 system (Agilent Technologies, Amstelveen, The Netherlands) was utilized to assess RNA quality and quantification was conducted using the ND-2000 Spectrophotometer (Thermo Inc., United States).

### 5.3 Library construction and sequencing

To obtain high-throughput transcriptome data from human liver cancer (Hep3B) cells, libraries were generated using the QuantSeq 3′mRNA-Seq Library Prep Kit (Lexogen, Inc., Austria) for both control and test RNAs, following the manufacturer’s instructions. Total RNA samples underwent reverse transcription with an oligo-dT primer that included an Illumina-compatible sequence at the 5′end. After degradation of the RNA template, second-strand synthesis was initiated using a random primer with an Illumina-compatible linker sequence at its 5′end. For the quality control step, magnetic bead purification was performed to remove reaction components from the double-stranded library. The library was then amplified to incorporate all necessary adaptor sequences for cluster generation, followed by purification to separate the final library from PCR components. High-throughput sequencing was conducted on the NextSeq 550 platform (Illumina, Inc., United States) with a read length of 75 bp, using single-end sequencing.

### 5.4 Data analysis

The raw reads obtained from sequencing underwent rigorous quality assessment and trimming procedures to ensure reliability and accuracy in subsequent analysis. Quality control measures were implemented, and reads were trimmed based on a threshold of greater than average Q20, utilizing the BBDuk tool. Trimmed reads underwent alignment utilizing Bowtie2 version 2.5.1 (Langmead and Salzberg, 2012). The resulting alignment file facilitated transcript assembly. Differentially expressed genes were identified based on counts obtained from unique and multiple alignments utilizing coverage in Bedtools v2.31.0 (Quinlan and Hall, 2010). Read Count (RC) data were subjected to processing via the TMM (Trimmed Mean of M values) + CPM (Counts per million) normalization method using EdgeR within the R environment (R Development Core Team, 2020) (Makowski et al., 2020). Genes exhibiting a log_2_-fold-change ≥ 1 and a false discovery rate (FDR) below 0.05 in pairwise comparison between two conditions were selected as differentially expressed genes. The log2FC threshold was set to ≥ 1 to ensure that the identified DEGs represent substantial changes in gene expression that are likely to be biologically relevant also FDR threshold of 0.05 was selected to control for multiple testing errors.

### 5.5 Gene ontology and KEGG analysis

Metascape version 3.5.20240101 (Zhou et al., 2019) (https://metascape.org/gp/index.html#/main/step1) is used as the computational tool for the prediction and clustering Gene Ontology (GO) terms of DEGs. The terms were categorized into biological processes, cellular compounds, and molecular functions. Additionally, for the Kyoto Encyclopedia of Genes and Genomes (KEGG) pathway analysis, the Pathview software (Luo et al., 2017) (https://pathview.uncc.edu/analysis) was employed, and the resulting pathways were visualized. Enrichment analysis for both GO terms and pathways was conducted with a significance threshold set at a *p*-value < 0.05. All the bubble plots in the manuscript were made from SR Plot (Tang et al., 2023) (https://www.bioinformatics.com.cn/srplot) based on the ggplot2 R package.

### 5.6 Functional annotation analysis

In the context of functional annotation analysis, the GeneCodis platform (https://genecodis.genyo.es/) (Garcia-Moreno et al., 2022) was employed to systematically explore the gene set. The criteria for selection included specifying *Homo sapiens* as the organism and using gene/protein identifiers as the input type. For the analysis, functional enrichment of GO terms was performed across three domains: Biological Process (GO BP), Cellular Component (GO CC), and Molecular Function (GO MF). The top 10 enriched terms were used to construct a gene-annotation cluster network. This analysis aimed to elucidate the functional significance of the identified genes, particularly in relation to their roles in biological processes, molecular functions, and cellular components.

### 5.7 Protein-protein interaction network construction

The PPI network for the common genes from the functional annotation network was sorted and the PPI network was constructed using STRING v12.0 (https://string-db.org/) with average local clustering coefficient of 0.9. The results from STRING were exported to Cytoscape v3.9.1. The Molecular Complex Detection (MCODE) plugin in Cytoscape V 3.9.1 (https://cytoscape.org/) was employed to identify densely interconnected clusters. The selection criteria were: degree ≥ 2, node score ≥ 0.2, K-core ≥ 2, and max depth = 100. For color gradient mcodeCluster–continuous mapping was applied with the range 1.0–3.0. The clustered networks were analysed using FunRich V 3.1.3 (http://www.funrich.org/) software for module construction and central node gene prediction.

### 5.8 Western blot analysis

Hep3B cells were seeded at a density of 5 × 10^4^ cells/plate to investigate protein expression. Cells were treated with 0 and 30 μM PUR for 24 h at 37°C. Subsequently, following cell harvesting, RIPA buffer (iNtRON Biotechnology, South Korea) was employed to lyse the cells. The protein concentration was determined using the Pierce™ BCA protein assay kit (Thermo Scientific). A total of 10 μg of protein was loaded and separated on SDS-PAGE. The gel was then transferred to PVDF membranes using a Semi-Dry Transfer machine (Atto-Corporation, Tokyo). Following blocking for one to 2 hours at room temperature, then membranes were incubated with diluted primary antibodies (1:1,000) at 4°C overnight. After washing with 1 × TBST solution, horseradish peroxidase-conjugated secondary antibodies (1:5,000) were used to probe the membranes for 2 h at room temperature (antibody dilutions were followed from standard protocols). Again blots were washed with 1 × TBST and developed using an ECL (electro-chemiluminescence) detection system (Bio-Rad Laboratory, United States). Protein densitometry was assessed using the ImageJ software program (NIH, United States). The data are validated using Bonferroni’s Multiple Comparison Test using GraphPad Prism V.5.01 statistical analysis software.

## Data Availability

The datasets presented in this study can be found in online repositories. The names of the repository/repositories and accession number(s) can be found below: https://www.ncbi.nlm.nih.gov/, PRJNA1083735.
