# Supplementary material for: Transcriptome analysis revealed the genes and major pathways involved in prunetrin treated hepatocellular carcinoma cells
Source: Front Pharmacol. 2024 Nov 1;15:1400186. doi: 10.3389/fphar.2024.1400186 (PMC11563786; doi:10.3389/fphar.2024.1400186)
Supplement: Supplementary file 1 [file DataSheet1.docx]

**Table S1.** Total sequencing read counts and statistics

| Name | Type | Reads | Bases | GC | N | Q30 |
| --- | --- | --- | --- | --- | --- | --- |
| Control | Raw | 16,103,601 (100%) | 1,223,873,676 (100%) | 536,113,369 (43.80%) | 11,201 (0.0%) | 1,137,366,596 (92.93%) |
|  | Clean | 15,313,043 (95.09%) | 928,170,741 (75.84%) | 403,351,492 (43.46%) | 2,286 (0.0%) | 879,143,989 (94.72%) |
| Treat | Raw | 15,543,456 (100%) | 1,181,302,656 (100%) | 514,878,237 (43.59%) | 10,979 (0.0%) | 1,101,111,138 (93.21%) |
|  | Clean | 14,849,777 (95.54%) | 907,997,122 (76.86%) | 391,525,476 (43.12%) | 2,204 (0.0%) | 860,013,714 (94.72%) |

^*^Q30: Number of over Q30 bases (Q30: 99.9% Base Call Accuracy) (Q30/Bases x100)

**Table S2.** Top 10 significantly enriched KEGG pathways

| **KEGG ID** | **Pathway Description** | **P value** | **Count** |
| --- | --- | --- | --- |
| hsa05200 | Pathways in cancer | 8.13E-05 | 122 |
| hsa04151 | PI3K-Akt signaling pathway | 6.58E-23 | 106 |
| hsa05165 | Human papillomavirus infection | 5.61E-23 | 98 |
| hsa05022 | Pathways of neurodegeneration - multiple diseases | 5.13E-05 | 89 |
| hsa04010 | MAPK signaling pathway | 4.04E-18 | 107 |
| hsa04110 | Cell cycle | 4.02E-18 | 93 |
| hsa05226 | Gastric cancer | 3.88E-19 | 57 |
| hsa05225 | Hepatocellular carcinoma | 3.46E-13 | 66 |
| hsa04210 | Apoptosis | 2.66E-17 | 108 |

**Table S3.** List of common genes from functional annotation and selected for the PPI network construction

| **S. No** | **Gene** | **S. No** | **Gene** |
| --- | --- | --- | --- |
| 1. | *DAB2IP* | 16. | *MYC* |
| 2. | *TNFSF10* | 17. | *TP53* |
| 3. | *BCL2L1* | 18. | *FAS* |
| 4. | *BID* | 19. | *AKT1* |
| 5. | *TGFB1* | 20. | *NFKB1* |
| 6. | *BAX* | 21. | *RAC1* |
| 7. | *TGFBR2* | 22. | *CCND1* |
| 8. | *TGFBR1* | 23. | *BCL2* |
| 9. | *PYCARD* | 24. | *BIRC5* |
| 10. | *CDKN2A* | 25. | *XIAP* |
| 11. | *FADD* | 26. | *BIRC2* |
| 12. | *VEGFA* | 27. | *CASP8* |
| 13. | *IGF2* | 28. | *MCL1* |
| 14. | *PTK2* | 29. | *TNFRSF10B* |
| 15. | *TGFB2* | 30. | *GSTP1* |

**
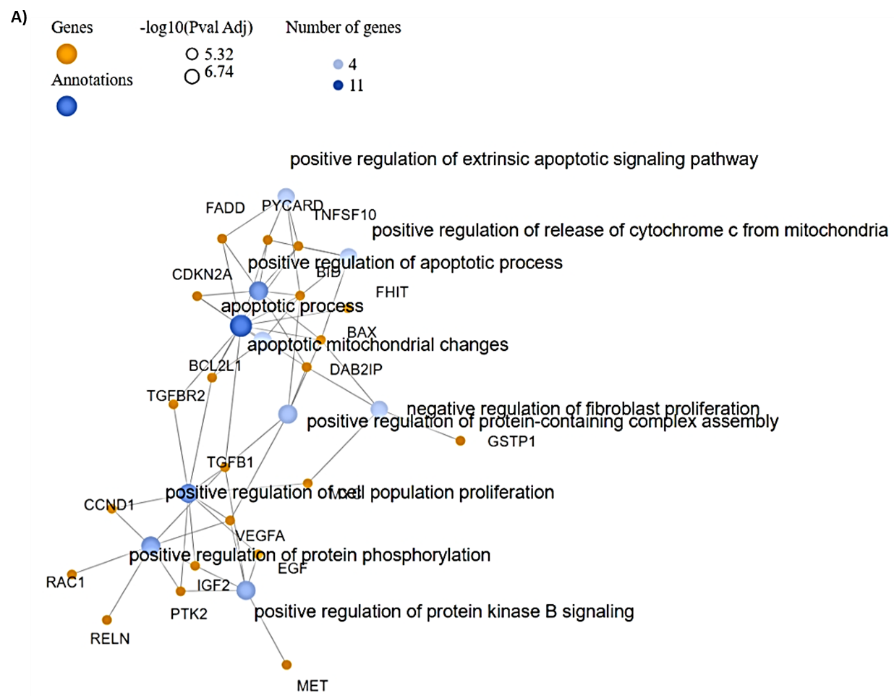
**

**
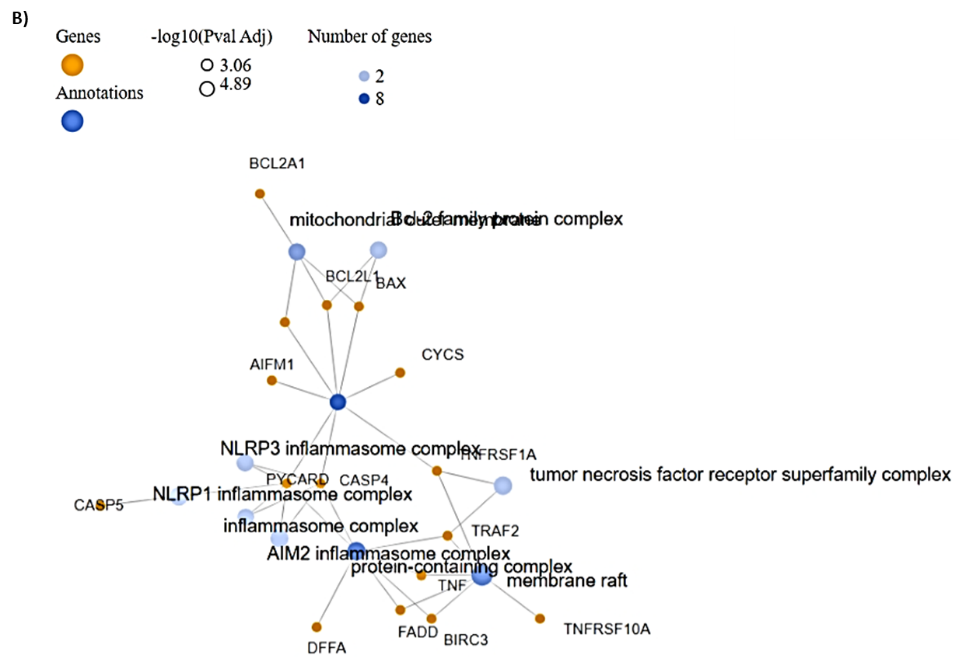
**

**
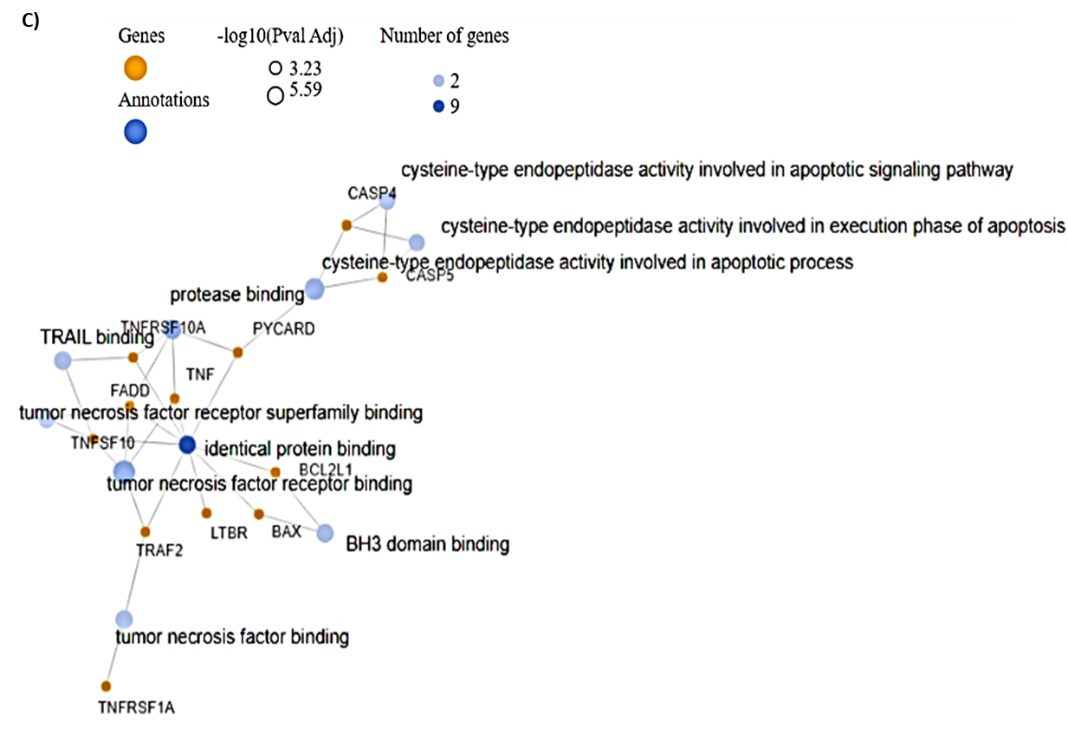
**

**Figure S1.** Functional annotation network construction. A) Biological process; B) Cellular components; C) Molecular function.

**
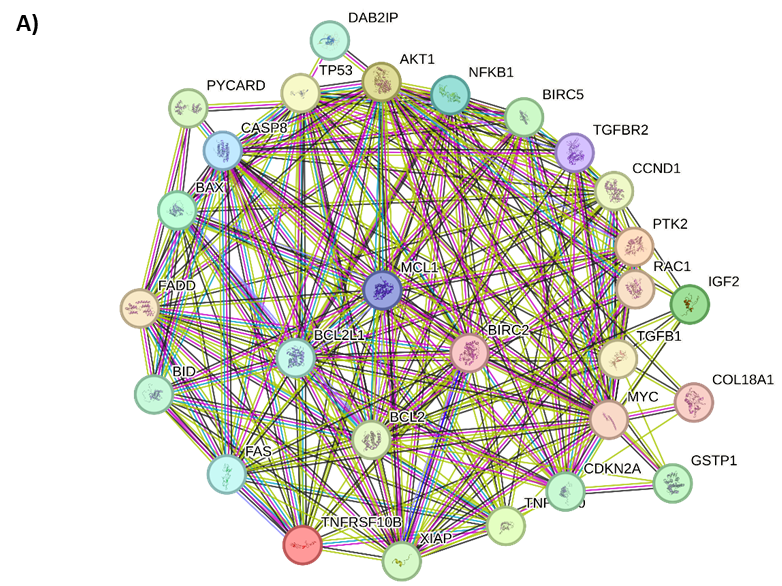
**

**Figure S2.** Protein-Protein Interaction (PPI) analysis conducted using the STRING database


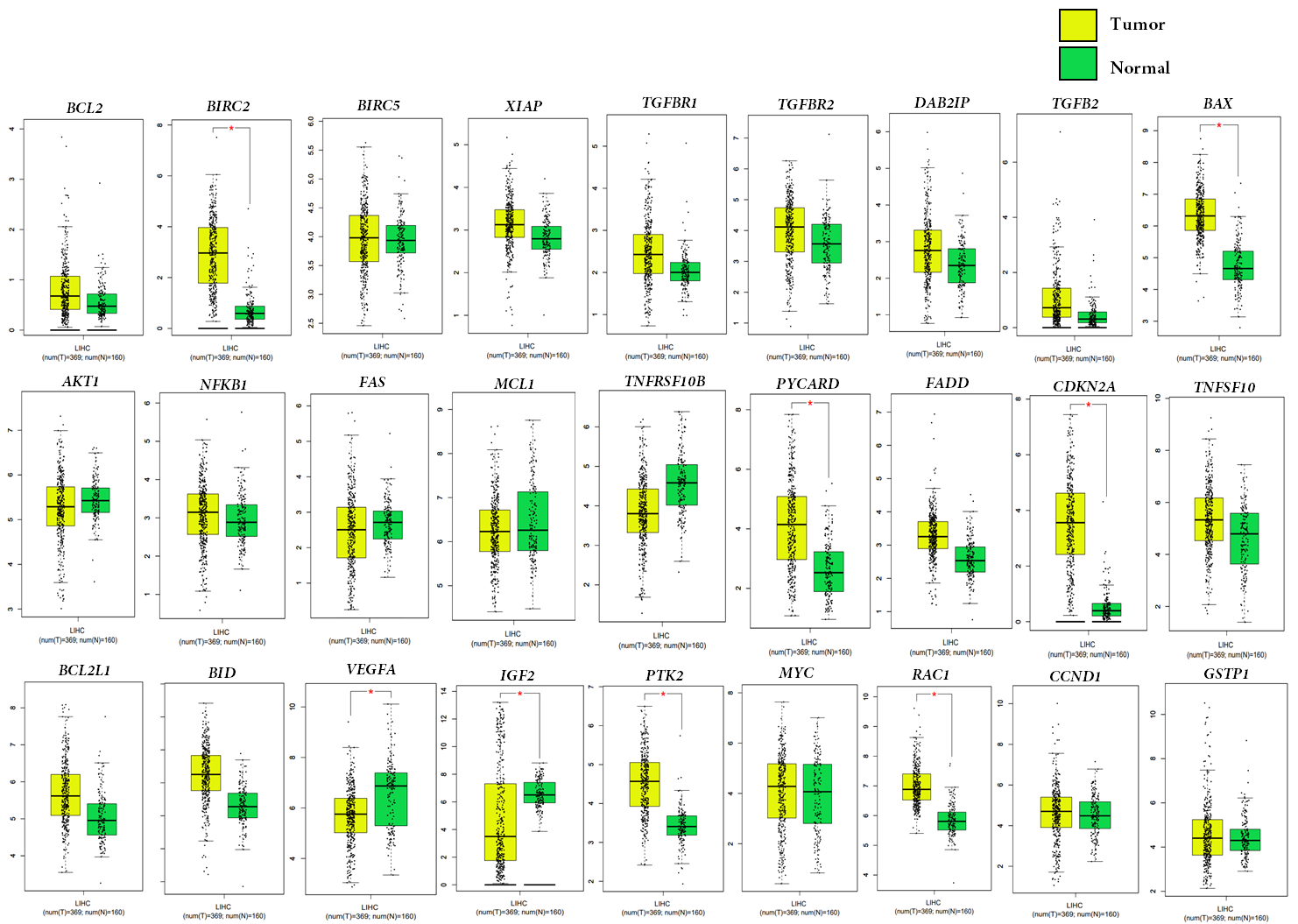


**Figure S3.** Box Plot analysis for the common genes from functional annotation analysis.

| **1** | ENSG00000001631 | **51** | ENSG00000077549 | **101** | ENSG00000105649 | **151** | ENSG00000116157 |
| --- | --- | --- | --- | --- | --- | --- | --- |
| **2** | ENSG00000002016 | **52** | ENSG00000077585 | **102** | ENSG00000105825 | **152** | ENSG00000116198 |
| **3** | ENSG00000004897 | **53** | ENSG00000077943 | **103** | ENSG00000105851 | **153** | ENSG00000116251 |
| **4** | ENSG00000004975 | **54** | ENSG00000079246 | **104** | ENSG00000105997 | **154** | ENSG00000116337 |
| **5** | ENSG00000005073 | **55** | ENSG00000079950 | **105** | ENSG00000106236 | **155** | ENSG00000116747 |
| **6** | ENSG00000006638 | **56** | ENSG00000080603 | **106** | ENSG00000106305 | **156** | ENSG00000116786 |
| **7** | ENSG00000008513 | **57** | ENSG00000080910 | **107** | ENSG00000106355 | **157** | ENSG00000116830 |
| **8** | ENSG00000009709 | **58** | ENSG00000081059 | **108** | ENSG00000107105 | **158** | ENSG00000117091 |
| **9** | ENSG00000010278 | **59** | ENSG00000083457 | **109** | ENSG00000107372 | **159** | ENSG00000117399 |
| **10** | ENSG00000010438 | **60** | ENSG00000084234 | **110** | ENSG00000107643 | **160** | ENSG00000117410 |
| **11** | ENSG00000011304 | **61** | ENSG00000085832 | **111** | ENSG00000107651 | **161** | ENSG00000117480 |
| **12** | ENSG00000013523 | **62** | ENSG00000086205 | **112** | ENSG00000108384 | **162** | ENSG00000118263 |
| **13** | ENSG00000015171 | **63** | ENSG00000087077 | **113** | ENSG00000108443 | **163** | ENSG00000118600 |
| **14** | ENSG00000018189 | **64** | ENSG00000087250 | **114** | ENSG00000108604 | **164** | ENSG00000119397 |
| **15** | ENSG00000025800 | **65** | ENSG00000088899 | **115** | ENSG00000108671 | **165** | ENSG00000120094 |
| **16** | ENSG00000026559 | **66** | ENSG00000089289 | **116** | ENSG00000108788 | **166** | ENSG00000120129 |
| **17** | ENSG00000029534 | **67** | ENSG00000089820 | **117** | ENSG00000108852 | **167** | ENSG00000120215 |
| **18** | ENSG00000033627 | **68** | ENSG00000091129 | **118** | ENSG00000109079 | **168** | ENSG00000120500 |
| **19** | ENSG00000033867 | **69** | ENSG00000091157 | **119** | ENSG00000109381 | **169** | ENSG00000120656 |
| **20** | ENSG00000039068 | **70** | ENSG00000092108 | **120** | ENSG00000109654 | **170** | ENSG00000121075 |
| **21** | ENSG00000044524 | **71** | ENSG00000092203 | **121** | ENSG00000109685 | **171** | ENSG00000121753 |
| **22** | ENSG00000052802 | **72** | ENSG00000093167 | **122** | ENSG00000109738 | **172** | ENSG00000122884 |
| **23** | ENSG00000057608 | **73** | ENSG00000095794 | **123** | ENSG00000110092 | **173** | ENSG00000123405 |
| **24** | ENSG00000062650 | **74** | ENSG00000099960 | **124** | ENSG00000110195 | **174** | ENSG00000123843 |
| **25** | ENSG00000063587 | **75** | ENSG00000100023 | **125** | ENSG00000110917 | **175** | ENSG00000124107 |
| **26** | ENSG00000064042 | **76** | ENSG00000100122 | **126** | ENSG00000111046 | **176** | ENSG00000124181 |
| **27** | ENSG00000064651 | **77** | ENSG00000100241 | **127** | ENSG00000111335 | **177** | ENSG00000124568 |
| **28** | ENSG00000064666 | **78** | ENSG00000100281 | **128** | ENSG00000111596 | **178** | ENSG00000124659 |
| **29** | ENSG00000064835 | **79** | ENSG00000100601 | **129** | ENSG00000111713 | **179** | ENSG00000124721 |
| **30** | ENSG00000064995 | **80** | ENSG00000100813 | **130** | ENSG00000111799 | **180** | ENSG00000125266 |
| **31** | ENSG00000064999 | **81** | ENSG00000100934 | **131** | ENSG00000111801 | **181** | ENSG00000125356 |
| **32** | ENSG00000065325 | **82** | ENSG00000101150 | **132** | ENSG00000111913 | **182** | ENSG00000125482 |
| **33** | ENSG00000065518 | **83** | ENSG00000101224 | **133** | ENSG00000112280 | **183** | ENSG00000125651 |
| **34** | ENSG00000065534 | **84** | ENSG00000101265 | **134** | ENSG00000112303 | **184** | ENSG00000125817 |
| **35** | ENSG00000065613 | **85** | ENSG00000101407 | **135** | ENSG00000112419 | **185** | ENSG00000125931 |
| **36** | ENSG00000067141 | **86** | ENSG00000101444 | **136** | ENSG00000112763 | **186** | ENSG00000126602 |
| **37** | ENSG00000067445 | **87** | ENSG00000101846 | **137** | ENSG00000113068 | **187** | ENSG00000127472 |
| **38** | ENSG00000068383 | **88** | ENSG00000102104 | **138** | ENSG00000113070 | **188** | ENSG00000127528 |
| **39** | ENSG00000068438 | **89** | ENSG00000102172 | **139** | ENSG00000113108 | **189** | ENSG00000127948 |
| **40** | ENSG00000069399 | **90** | ENSG00000102678 | **140** | ENSG00000113494 | **190** | ENSG00000127955 |
| **41** | ENSG00000069974 | **91** | ENSG00000102755 | **141** | ENSG00000113569 | **191** | ENSG00000128271 |
| **42** | ENSG00000072736 | **92** | ENSG00000103343 | **142** | ENSG00000114388 | **192** | ENSG00000128276 |
| **43** | ENSG00000074219 | **93** | ENSG00000103507 | **143** | ENSG00000114480 | **193** | ENSG00000128285 |
| **44** | ENSG00000075624 | **94** | ENSG00000104415 | **144** | ENSG00000114520 | **194** | ENSG00000128512 |
| **45** | ENSG00000075651 | **95** | ENSG00000104833 | **145** | ENSG00000114784 | **195** | ENSG00000128591 |
| **46** | ENSG00000075914 | **96** | ENSG00000104969 | **146** | ENSG00000115339 | **196** | ENSG00000128656 |
| **47** | ENSG00000076258 | **97** | ENSG00000105146 | **147** | ENSG00000115504 | **197** | ENSG00000128708 |
| **48** | ENSG00000076554 | **98** | ENSG00000105223 | **148** | ENSG00000115718 | **198** | ENSG00000129167 |
| **49** | ENSG00000077380 | **99** | ENSG00000105388 | **149** | ENSG00000115884 | **199** | ENSG00000129219 |
| **50** | ENSG00000077498 | **100** | ENSG00000105409 | **150** | ENSG00000116044 | **200** | ENSG00000130222 |

**List of Up-regulated DEGs**

| **201** | ENSG00000130226 | **251** | ENSG00000140391 | **301** | ENSG00000156219 | **351** | ENSG00000169330 |
| --- | --- | --- | --- | --- | --- | --- | --- |
| **202** | ENSG00000130294 | **252** | ENSG00000140521 | **302** | ENSG00000156467 | **352** | ENSG00000169676 |
| **203** | ENSG00000130513 | **253** | ENSG00000140575 | **303** | ENSG00000156885 | **353** | ENSG00000169715 |
| **204** | ENSG00000131096 | **254** | ENSG00000140682 | **304** | ENSG00000158560 | **354** | ENSG00000170122 |
| **205** | ENSG00000131437 | **255** | ENSG00000141198 | **305** | ENSG00000158869 | **355** | ENSG00000170606 |
| **206** | ENSG00000131910 | **256** | ENSG00000141506 | **306** | ENSG00000159110 | **356** | ENSG00000170759 |
| **207** | ENSG00000132341 | **257** | ENSG00000141736 | **307** | ENSG00000159450 | **357** | ENSG00000170965 |
| **208** | ENSG00000132356 | **258** | ENSG00000141959 | **308** | ENSG00000160223 | **358** | ENSG00000171150 |
| **209** | ENSG00000132361 | **259** | ENSG00000142731 | **309** | ENSG00000160294 | **359** | ENSG00000171530 |
| **210** | ENSG00000132470 | **260** | ENSG00000142867 | **310** | ENSG00000160310 | **360** | ENSG00000171700 |
| **211** | ENSG00000132604 | **261** | ENSG00000143013 | **311** | ENSG00000160752 | **361** | ENSG00000171703 |
| **212** | ENSG00000132639 | **262** | ENSG00000143061 | **312** | ENSG00000161057 | **362** | ENSG00000172059 |
| **213** | ENSG00000133216 | **263** | ENSG00000143119 | **313** | ENSG00000161202 | **363** | ENSG00000172179 |
| **214** | ENSG00000133226 | **264** | ENSG00000143252 | **314** | ENSG00000162399 | **364** | ENSG00000172262 |
| **215** | ENSG00000133800 | **265** | ENSG00000143321 | **315** | ENSG00000162591 | **365** | ENSG00000172493 |
| **216** | ENSG00000134200 | **266** | ENSG00000143847 | **316** | ENSG00000162736 | **366** | ENSG00000173531 |
| **217** | ENSG00000134248 | **267** | ENSG00000144021 | **317** | ENSG00000163159 | **367** | ENSG00000173852 |
| **218** | ENSG00000134480 | **268** | ENSG00000145020 | **318** | ENSG00000163273 | **368** | ENSG00000174255 |
| **219** | ENSG00000134516 | **269** | ENSG00000145217 | **319** | ENSG00000164086 | **369** | ENSG00000174851 |
| **220** | ENSG00000134545 | **270** | ENSG00000145242 | **320** | ENSG00000164111 | **370** | ENSG00000174876 |
| **221** | ENSG00000134755 | **271** | ENSG00000145864 | **321** | ENSG00000164128 | **371** | ENSG00000175220 |
| **222** | ENSG00000134827 | **272** | ENSG00000146457 | **322** | ENSG00000164543 | **372** | ENSG00000175336 |
| **223** | ENSG00000134910 | **273** | ENSG00000146592 | **323** | ENSG00000164754 | **373** | ENSG00000175390 |
| **224** | ENSG00000135164 | **274** | ENSG00000147145 | **324** | ENSG00000164930 | **374** | ENSG00000175591 |
| **225** | ENSG00000135298 | **275** | ENSG00000147394 | **325** | ENSG00000165376 | **375** | ENSG00000175906 |
| **226** | ENSG00000135404 | **276** | ENSG00000147432 | **326** | ENSG00000165416 | **376** | ENSG00000176171 |
| **227** | ENSG00000135624 | **277** | ENSG00000147862 | **327** | ENSG00000165556 | **377** | ENSG00000176406 |
| **228** | ENSG00000135828 | **278** | ENSG00000148180 | **328** | ENSG00000165630 | **378** | ENSG00000177613 |
| **229** | ENSG00000135902 | **279** | ENSG00000148671 | **329** | ENSG00000165699 | **379** | ENSG00000177663 |
| **230** | ENSG00000135966 | **280** | ENSG00000149131 | **330** | ENSG00000166037 | **380** | ENSG00000178035 |
| **231** | ENSG00000136160 | **281** | ENSG00000149591 | **331** | ENSG00000166073 | **381** | ENSG00000178363 |
| **232** | ENSG00000137090 | **282** | ENSG00000150337 | **332** | ENSG00000166347 | **382** | ENSG00000179772 |
| **233** | ENSG00000137193 | **283** | ENSG00000151503 | **333** | ENSG00000166483 | **383** | ENSG00000179915 |
| **234** | ENSG00000137252 | **284** | ENSG00000151617 | **334** | ENSG00000166787 | **384** | ENSG00000180817 |
| **235** | ENSG00000137270 | **285** | ENSG00000151651 | **335** | ENSG00000166888 | **385** | ENSG00000181418 |
| **236** | ENSG00000137392 | **286** | ENSG00000152669 | **336** | ENSG00000167004 | **386** | ENSG00000181562 |
| **237** | ENSG00000137491 | **287** | ENSG00000152822 | **337** | ENSG00000167114 | **387** | ENSG00000181965 |
| **238** | ENSG00000137575 | **288** | ENSG00000153132 | **338** | ENSG00000167286 | **388** | ENSG00000182253 |
| **239** | ENSG00000137673 | **289** | ENSG00000153233 | **339** | ENSG00000167434 | **389** | ENSG00000182473 |
| **240** | ENSG00000137802 | **290** | ENSG00000153283 | **340** | ENSG00000167635 | **390** | ENSG00000182985 |
| **241** | ENSG00000138311 | **291** | ENSG00000153487 | **341** | ENSG00000167657 | **391** | ENSG00000183155 |
| **242** | ENSG00000138448 | **292** | ENSG00000153923 | **342** | ENSG00000167815 | **392** | ENSG00000183161 |
| **243** | ENSG00000138472 | **293** | ENSG00000154016 | **343** | ENSG00000167969 | **393** | ENSG00000183580 |
| **244** | ENSG00000138615 | **294** | ENSG00000154263 | **344** | ENSG00000168283 | **394** | ENSG00000183628 |
| **245** | ENSG00000138772 | **295** | ENSG00000154646 | **345** | ENSG00000168385 | **395** | ENSG00000183668 |
| **246** | ENSG00000138798 | **296** | ENSG00000154678 | **346** | ENSG00000168393 | **396** | ENSG00000183684 |
| **247** | ENSG00000139193 | **297** | ENSG00000154928 | **347** | ENSG00000168397 | **397** | ENSG00000183696 |
| **248** | ENSG00000139546 | **298** | ENSG00000155087 | **348** | ENSG00000168546 | **398** | ENSG00000183963 |
| **249** | ENSG00000139547 | **299** | ENSG00000155506 | **349** | ENSG00000169297 | **399** | ENSG00000184110 |
| **250** | ENSG00000140262 | **300** | ENSG00000156140 | **350** | ENSG00000169306 | **400** | ENSG00000184349 |

| **401** | ENSG00000184445 | **451** | ENSG00000228278 |
| --- | --- | --- | --- |
| **402** | ENSG00000185483 | **452** | ENSG00000230675 |
| **403** | ENSG00000185697 | **453** | ENSG00000230995 |
| **404** | ENSG00000185813 | **454** | ENSG00000231924 |
| **405** | ENSG00000186297 | **455** | ENSG00000233816 |
| **406** | ENSG00000186376 | **456** | ENSG00000236824 |
| **407** | ENSG00000186642 | **457** | ENSG00000236843 |
| **408** | ENSG00000186832 | **458** | ENSG00000239305 |
| **409** | ENSG00000187323 | **459** | ENSG00000239672 |
| **410** | ENSG00000187486 | **460** | ENSG00000243725 |
| **411** | ENSG00000187908 | **461** | ENSG00000244462 |
| **412** | ENSG00000188130 | **462** | ENSG00000248848 |
| **413** | ENSG00000188641 | **463** | ENSG00000251493 |
| **414** | ENSG00000189091 | **464** | ENSG00000256087 |
| **415** | ENSG00000196091 | **465** | ENSG00000256612 |
| **416** | ENSG00000196418 | **466** | ENSG00000257138 |
| **417** | ENSG00000196420 | **467** | ENSG00000257267 |
| **418** | ENSG00000196576 | **468** | ENSG00000260873 |
| **419** | ENSG00000196639 | **469** | ENSG00000261609 |
| **420** | ENSG00000196843 | **470** | ENSG00000261857 |
| **421** | ENSG00000196866 | **471** | ENSG00000263290 |
| **422** | ENSG00000196976 | **472** | ENSG00000267534 |
| **423** | ENSG00000197008 | **473** | ENSG00000273993 |
| **424** | ENSG00000197724 | **474** | ENSG00000274324 |
| **425** | ENSG00000197780 | **475** | ENSG00000274542 |
| **426** | ENSG00000197961 | **476** | ENSG00000274665 |
| **427** | ENSG00000198125 | **477** | ENSG00000276114 |
| **428** | ENSG00000198198 | **478** | ENSG00000276482 |
| **429** | ENSG00000198205 | **479** | ENSG00000276676 |
| **430** | ENSG00000198216 | **480** | ENSG00000276734 |
| **431** | ENSG00000198336 | **481** | ENSG00000276825 |
| **432** | ENSG00000198420 | **482** | ENSG00000280858 |
| **433** | ENSG00000198695 | **483** | ENSG00000280908 |
| **434** | ENSG00000198774 | **484** | ENSG00000281306 |
| **435** | ENSG00000198821 | **485** | ENSG00000281484 |
| **436** | ENSG00000198952 | **486** | ENSG00000281708 |
| **437** | ENSG00000198963 | **487** | ENSG00000281766 |
| **438** | ENSG00000204628 | **488** | ENSG00000281857 |
| **439** | ENSG00000205220 | **489** | ENSG00000281886 |
| **440** | ENSG00000205364 | **490** | ENSG00000282941 |
| **441** | ENSG00000205542 | **491** | ENSG00000282984 |
| **442** | ENSG00000205937 | **492** | ENSG00000283046 |
| **443** | ENSG00000206561 | **493** | ENSG00000284807 |
| **444** | ENSG00000213218 | **494** | ENSG00000285434 |
| **445** | ENSG00000213462 | **495** | ENSG00000288121 |
| **446** | ENSG00000213465 | **496** | ENSG00000288169 |
| **447** | ENSG00000213927 | **497** | ENSG00000288217 |
| **448** | ENSG00000213949 | **498** | ENSG00000288307 |
| **449** | ENSG00000226071 | **499** | ENSG00000288485 |
| **450** | ENSG00000226651 |  |  |

**List of Down-regulated DEGs**

| **1** | ENSG00000002586 | **51** | ENSG00000074181 | **101** | ENSG00000101040 | **151** | ENSG00000108946 |
| --- | --- | --- | --- | --- | --- | --- | --- |
| **2** | ENSG00000004059 | **52** | ENSG00000074621 | **102** | ENSG00000101160 | **152** | ENSG00000108960 |
| **3** | ENSG00000005007 | **53** | ENSG00000075239 | **103** | ENSG00000101191 | **153** | ENSG00000109062 |
| **4** | ENSG00000008394 | **54** | ENSG00000075391 | **104** | ENSG00000101213 | **154** | ENSG00000109063 |
| **5** | ENSG00000008405 | **55** | ENSG00000075399 | **105** | ENSG00000101310 | **155** | ENSG00000109158 |
| **6** | ENSG00000008517 | **56** | ENSG00000076067 | **106** | ENSG00000101333 | **156** | ENSG00000109321 |
| **7** | ENSG00000011465 | **57** | ENSG00000076108 | **107** | ENSG00000101384 | **157** | ENSG00000109511 |
| **8** | ENSG00000012211 | **58** | ENSG00000076242 | **108** | ENSG00000101773 | **158** | ENSG00000109736 |
| **9** | ENSG00000013375 | **59** | ENSG00000076382 | **109** | ENSG00000101782 | **159** | ENSG00000109756 |
| **10** | ENSG00000013588 | **60** | ENSG00000079134 | **110** | ENSG00000101892 | **160** | ENSG00000110046 |
| **11** | ENSG00000018510 | **61** | ENSG00000079689 | **111** | ENSG00000102466 | **161** | ENSG00000110076 |
| **12** | ENSG00000019186 | **62** | ENSG00000080493 | **112** | ENSG00000102738 | **162** | ENSG00000110344 |
| **13** | ENSG00000021852 | **63** | ENSG00000080709 | **113** | ENSG00000102970 | **163** | ENSG00000110400 |
| **14** | ENSG00000027075 | **64** | ENSG00000081051 | **114** | ENSG00000103024 | **164** | ENSG00000110492 |
| **15** | ENSG00000031698 | **65** | ENSG00000083312 | **115** | ENSG00000103266 | **165** | ENSG00000110514 |
| **16** | ENSG00000035681 | **66** | ENSG00000083799 | **116** | ENSG00000103647 | **166** | ENSG00000110697 |
| **17** | ENSG00000035928 | **67** | ENSG00000083857 | **117** | ENSG00000103995 | **167** | ENSG00000110811 |
| **18** | ENSG00000036473 | **68** | ENSG00000084453 | **118** | ENSG00000104131 | **168** | ENSG00000110852 |
| **19** | ENSG00000036828 | **69** | ENSG00000084623 | **119** | ENSG00000104313 | **169** | ENSG00000110876 |
| **20** | ENSG00000039537 | **70** | ENSG00000084674 | **120** | ENSG00000104435 | **170** | ENSG00000110880 |
| **21** | ENSG00000039600 | **71** | ENSG00000085276 | **121** | ENSG00000104695 | **171** | ENSG00000110887 |
| **22** | ENSG00000042429 | **72** | ENSG00000085871 | **122** | ENSG00000104899 | **172** | ENSG00000111432 |
| **23** | ENSG00000047849 | **73** | ENSG00000086061 | **123** | ENSG00000105135 | **173** | ENSG00000111725 |
| **24** | ENSG00000049192 | **74** | ENSG00000086570 | **124** | ENSG00000105205 | **174** | ENSG00000111737 |
| **25** | ENSG00000049245 | **75** | ENSG00000087237 | **125** | ENSG00000105329 | **175** | ENSG00000112062 |
| **26** | ENSG00000051620 | **76** | ENSG00000090060 | **126** | ENSG00000105355 | **176** | ENSG00000112130 |
| **27** | ENSG00000053524 | **77** | ENSG00000091651 | **127** | ENSG00000105401 | **177** | ENSG00000112175 |
| **28** | ENSG00000054277 | **78** | ENSG00000091879 | **128** | ENSG00000105464 | **178** | ENSG00000112319 |
| **29** | ENSG00000054598 | **79** | ENSG00000092820 | **129** | ENSG00000105656 | **179** | ENSG00000112655 |
| **30** | ENSG00000058063 | **80** | ENSG00000096395 | **130** | ENSG00000105722 | **180** | ENSG00000112706 |
| **31** | ENSG00000058335 | **81** | ENSG00000099889 | **131** | ENSG00000105993 | **181** | ENSG00000112715 |
| **32** | ENSG00000059728 | **82** | ENSG00000099968 | **132** | ENSG00000106038 | **182** | ENSG00000112941 |
| **33** | ENSG00000061337 | **83** | ENSG00000099995 | **133** | ENSG00000106113 | **183** | ENSG00000113163 |
| **34** | ENSG00000062822 | **84** | ENSG00000100030 | **134** | ENSG00000106536 | **184** | ENSG00000113552 |
| **35** | ENSG00000065243 | **85** | ENSG00000100060 | **135** | ENSG00000106615 | **185** | ENSG00000114209 |
| **36** | ENSG00000065357 | **86** | ENSG00000100077 | **136** | ENSG00000106665 | **186** | ENSG00000114316 |
| **37** | ENSG00000065427 | **87** | ENSG00000100142 | **137** | ENSG00000107018 | **187** | ENSG00000114554 |
| **38** | ENSG00000066027 | **88** | ENSG00000100243 | **138** | ENSG00000107201 | **188** | ENSG00000114573 |
| **39** | ENSG00000067992 | **89** | ENSG00000100292 | **139** | ENSG00000107331 | **189** | ENSG00000114859 |
| **40** | ENSG00000068323 | **90** | ENSG00000100307 | **140** | ENSG00000107521 | **190** | ENSG00000115155 |
| **41** | ENSG00000068354 | **91** | ENSG00000100368 | **141** | ENSG00000107554 | **191** | ENSG00000115641 |
| **42** | ENSG00000069431 | **92** | ENSG00000100373 | **142** | ENSG00000107611 | **192** | ENSG00000115750 |
| **43** | ENSG00000069943 | **93** | ENSG00000100387 | **143** | ENSG00000108055 | **193** | ENSG00000115896 |
| **44** | ENSG00000070182 | **94** | ENSG00000100426 | **144** | ENSG00000108342 | **194** | ENSG00000116062 |
| **45** | ENSG00000070444 | **95** | ENSG00000100478 | **145** | ENSG00000108349 | **195** | ENSG00000116128 |
| **46** | ENSG00000070601 | **96** | ENSG00000100577 | **146** | ENSG00000108395 | **196** | ENSG00000116489 |
| **47** | ENSG00000071054 | **97** | ENSG00000100578 | **147** | ENSG00000108759 | **197** | ENSG00000116701 |
| **48** | ENSG00000071564 | **98** | ENSG00000100591 | **148** | ENSG00000108786 | **198** | ENSG00000116824 |
| **49** | ENSG00000073711 | **99** | ENSG00000100867 | **149** | ENSG00000108797 | **199** | ENSG00000117090 |
| **50** | ENSG00000073737 | **100** | ENSG00000101003 | **150** | ENSG00000108883 | **200** | ENSG00000117152 |

| **201** | ENSG00000117411 | **251** | ENSG00000127920 | **301** | ENSG00000135407 | **351** | ENSG00000143365 |
| --- | --- | --- | --- | --- | --- | --- | --- |
| **202** | ENSG00000117620 | **252** | ENSG00000128059 | **302** | ENSG00000135409 | **352** | ENSG00000143384 |
| **203** | ENSG00000117707 | **253** | ENSG00000128567 | **303** | ENSG00000135486 | **353** | ENSG00000143434 |
| **204** | ENSG00000117906 | **254** | ENSG00000128590 | **304** | ENSG00000135517 | **354** | ENSG00000143537 |
| **205** | ENSG00000118058 | **255** | ENSG00000128617 | **305** | ENSG00000135547 | **355** | ENSG00000143669 |
| **206** | ENSG00000118298 | **256** | ENSG00000128989 | **306** | ENSG00000135740 | **356** | ENSG00000143799 |
| **207** | ENSG00000118777 | **257** | ENSG00000129152 | **307** | ENSG00000135862 | **357** | ENSG00000143995 |
| **208** | ENSG00000119013 | **258** | ENSG00000129455 | **308** | ENSG00000135899 | **358** | ENSG00000144048 |
| **209** | ENSG00000119673 | **259** | ENSG00000129484 | **309** | ENSG00000135930 | **359** | ENSG00000144191 |
| **210** | ENSG00000119729 | **260** | ENSG00000130203 | **310** | ENSG00000136098 | **360** | ENSG00000144681 |
| **211** | ENSG00000119922 | **261** | ENSG00000130303 | **311** | ENSG00000136153 | **361** | ENSG00000144713 |
| **212** | ENSG00000119969 | **262** | ENSG00000130429 | **312** | ENSG00000136240 | **362** | ENSG00000145675 |
| **213** | ENSG00000120251 | **263** | ENSG00000130477 | **313** | ENSG00000136352 | **363** | ENSG00000147202 |
| **214** | ENSG00000120693 | **264** | ENSG00000130640 | **314** | ENSG00000136518 | **364** | ENSG00000147437 |
| **215** | ENSG00000120708 | **265** | ENSG00000130649 | **315** | ENSG00000136826 | **365** | ENSG00000147669 |
| **216** | ENSG00000120896 | **266** | ENSG00000130706 | **316** | ENSG00000136854 | **366** | ENSG00000148834 |
| **217** | ENSG00000120910 | **267** | ENSG00000130707 | **317** | ENSG00000136997 | **367** | ENSG00000149100 |
| **218** | ENSG00000121058 | **268** | ENSG00000130714 | **318** | ENSG00000137055 | **368** | ENSG00000149269 |
| **219** | ENSG00000121060 | **269** | ENSG00000130725 | **319** | ENSG00000137076 | **369** | ENSG00000149295 |
| **220** | ENSG00000121671 | **270** | ENSG00000130755 | **320** | ENSG00000137100 | **370** | ENSG00000149506 |
| **221** | ENSG00000121858 | **271** | ENSG00000130830 | **321** | ENSG00000137265 | **371** | ENSG00000149930 |
| **222** | ENSG00000121989 | **272** | ENSG00000131183 | **322** | ENSG00000137674 | **372** | ENSG00000150625 |
| **223** | ENSG00000122359 | **273** | ENSG00000131196 | **323** | ENSG00000137693 | **373** | ENSG00000150687 |
| **224** | ENSG00000122641 | **274** | ENSG00000131808 | **324** | ENSG00000137815 | **374** | ENSG00000150768 |
| **225** | ENSG00000122733 | **275** | ENSG00000131979 | **325** | ENSG00000137955 | **375** | ENSG00000150967 |
| **226** | ENSG00000122863 | **276** | ENSG00000132109 | **326** | ENSG00000137992 | **376** | ENSG00000151292 |
| **227** | ENSG00000122971 | **277** | ENSG00000132128 | **327** | ENSG00000138078 | **377** | ENSG00000151490 |
| **228** | ENSG00000123124 | **278** | ENSG00000132141 | **328** | ENSG00000138398 | **378** | ENSG00000151623 |
| **229** | ENSG00000123191 | **279** | ENSG00000132259 | **329** | ENSG00000138640 | **379** | ENSG00000152127 |
| **230** | ENSG00000123388 | **280** | ENSG00000132463 | **330** | ENSG00000139330 | **380** | ENSG00000152208 |
| **231** | ENSG00000123415 | **281** | ENSG00000132514 | **331** | ENSG00000139352 | **381** | ENSG00000152229 |
| **232** | ENSG00000123562 | **282** | ENSG00000132664 | **332** | ENSG00000139644 | **382** | ENSG00000152578 |
| **233** | ENSG00000123595 | **283** | ENSG00000132693 | **333** | ENSG00000140030 | **383** | ENSG00000152804 |
| **234** | ENSG00000123836 | **284** | ENSG00000132849 | **334** | ENSG00000140259 | **384** | ENSG00000153071 |
| **235** | ENSG00000123999 | **285** | ENSG00000132938 | **335** | ENSG00000140368 | **385** | ENSG00000153140 |
| **236** | ENSG00000124151 | **286** | ENSG00000132952 | **336** | ENSG00000140538 | **386** | ENSG00000153162 |
| **237** | ENSG00000124159 | **287** | ENSG00000133104 | **337** | ENSG00000140612 | **387** | ENSG00000153904 |
| **238** | ENSG00000124260 | **288** | ENSG00000133110 | **338** | ENSG00000141012 | **388** | ENSG00000154096 |
| **239** | ENSG00000124313 | **289** | ENSG00000133111 | **339** | ENSG00000141338 | **389** | ENSG00000154258 |
| **240** | ENSG00000124356 | **290** | ENSG00000133124 | **340** | ENSG00000141448 | **390** | ENSG00000154310 |
| **241** | ENSG00000125144 | **291** | ENSG00000133895 | **341** | ENSG00000141524 | **391** | ENSG00000155363 |
| **242** | ENSG00000125337 | **292** | ENSG00000133961 | **342** | ENSG00000142163 | **392** | ENSG00000155622 |
| **243** | ENSG00000125384 | **293** | ENSG00000133997 | **343** | ENSG00000142166 | **393** | ENSG00000155659 |
| **244** | ENSG00000125388 | **294** | ENSG00000134138 | **344** | ENSG00000142168 | **394** | ENSG00000156049 |
| **245** | ENSG00000125618 | **295** | ENSG00000134256 | **345** | ENSG00000142453 | **395** | ENSG00000156127 |
| **246** | ENSG00000125733 | **296** | ENSG00000134812 | **346** | ENSG00000142511 | **396** | ENSG00000156136 |
| **247** | ENSG00000125810 | **297** | ENSG00000134873 | **347** | ENSG00000142599 | **397** | ENSG00000156471 |
| **248** | ENSG00000126583 | **298** | ENSG00000134940 | **348** | ENSG00000142733 | **398** | ENSG00000156931 |
| **249** | ENSG00000126752 | **299** | ENSG00000135047 | **349** | ENSG00000142892 | **399** | ENSG00000156970 |
| **250** | ENSG00000126803 | **300** | ENSG00000135363 | **350** | ENSG00000143322 | **400** | ENSG00000157005 |

| **401** | ENSG00000157077 | **451** | ENSG00000167193 | **501** | ENSG00000174780 | **551** | ENSG00000189058 |
| --- | --- | --- | --- | --- | --- | --- | --- |
| **402** | ENSG00000157103 | **452** | ENSG00000167232 | **502** | ENSG00000175040 | **552** | ENSG00000189060 |
| **403** | ENSG00000157110 | **453** | ENSG00000167531 | **503** | ENSG00000175063 | **553** | ENSG00000189221 |
| **404** | ENSG00000157823 | **454** | ENSG00000167656 | **504** | ENSG00000175535 | **554** | ENSG00000196352 |
| **405** | ENSG00000157916 | **455** | ENSG00000167658 | **505** | ENSG00000175538 | **555** | ENSG00000196431 |
| **406** | ENSG00000158406 | **456** | ENSG00000167825 | **506** | ENSG00000175646 | **556** | ENSG00000196628 |
| **407** | ENSG00000159197 | **457** | ENSG00000167972 | **507** | ENSG00000176124 | **557** | ENSG00000196747 |
| **408** | ENSG00000159640 | **458** | ENSG00000168124 | **508** | ENSG00000176919 | **558** | ENSG00000196961 |
| **409** | ENSG00000159674 | **459** | ENSG00000168229 | **509** | ENSG00000177047 | **559** | ENSG00000197405 |
| **410** | ENSG00000160182 | **460** | ENSG00000168306 | **510** | ENSG00000177239 | **560** | ENSG00000197406 |
| **411** | ENSG00000160207 | **461** | ENSG00000168309 | **511** | ENSG00000178386 | **561** | ENSG00000197408 |
| **412** | ENSG00000160209 | **462** | ENSG00000168556 | **512** | ENSG00000178522 | **562** | ENSG00000197540 |
| **413** | ENSG00000160683 | **463** | ENSG00000168582 | **513** | ENSG00000178726 | **563** | ENSG00000197548 |
| **414** | ENSG00000160868 | **464** | ENSG00000168653 | **514** | ENSG00000178921 | **564** | ENSG00000197576 |
| **415** | ENSG00000160882 | **465** | ENSG00000168734 | **515** | ENSG00000179115 | **565** | ENSG00000197894 |
| **416** | ENSG00000160951 | **466** | ENSG00000168795 | **516** | ENSG00000179142 | **566** | ENSG00000197965 |
| **417** | ENSG00000162402 | **467** | ENSG00000169100 | **517** | ENSG00000179909 | **567** | ENSG00000198356 |
| **418** | ENSG00000162419 | **468** | ENSG00000169245 | **518** | ENSG00000180104 | **568** | ENSG00000198363 |
| **419** | ENSG00000162496 | **469** | ENSG00000169255 | **519** | ENSG00000180182 | **569** | ENSG00000198373 |
| **420** | ENSG00000162630 | **470** | ENSG00000169372 | **520** | ENSG00000180340 | **570** | ENSG00000198610 |
| **421** | ENSG00000162894 | **471** | ENSG00000169398 | **521** | ENSG00000180509 | **571** | ENSG00000198625 |
| **422** | ENSG00000163064 | **472** | ENSG00000169413 | **522** | ENSG00000180660 | **572** | ENSG00000198646 |
| **423** | ENSG00000163110 | **473** | ENSG00000169679 | **523** | ENSG00000182333 | **573** | ENSG00000198670 |
| **424** | ENSG00000163131 | **474** | ENSG00000169813 | **524** | ENSG00000182481 | **574** | ENSG00000198752 |
| **425** | ENSG00000163171 | **475** | ENSG00000170160 | **525** | ENSG00000182511 | **575** | ENSG00000198931 |
| **426** | ENSG00000163206 | **476** | ENSG00000170296 | **526** | ENSG00000182606 | **576** | ENSG00000203737 |
| **427** | ENSG00000163399 | **477** | ENSG00000170365 | **527** | ENSG00000183134 | **577** | ENSG00000205339 |
| **428** | ENSG00000163479 | **478** | ENSG00000170442 | **528** | ENSG00000183281 | **578** | ENSG00000205426 |
| **429** | ENSG00000163568 | **479** | ENSG00000170498 | **529** | ENSG00000183648 | **579** | ENSG00000211456 |
| **430** | ENSG00000163914 | **480** | ENSG00000170579 | **530** | ENSG00000184160 | **580** | ENSG00000213759 |
| **431** | ENSG00000163950 | **481** | ENSG00000171033 | **531** | ENSG00000184185 | **581** | ENSG00000213931 |
| **432** | ENSG00000164022 | **482** | ENSG00000171051 | **532** | ENSG00000184209 | **582** | ENSG00000214274 |
| **433** | ENSG00000164106 | **483** | ENSG00000171360 | **533** | ENSG00000184895 | **583** | ENSG00000215788 |
| **434** | ENSG00000164109 | **484** | ENSG00000171368 | **534** | ENSG00000184979 | **584** | ENSG00000221819 |
| **435** | ENSG00000164163 | **485** | ENSG00000171388 | **535** | ENSG00000185338 | **585** | ENSG00000221823 |
| **436** | ENSG00000164626 | **486** | ENSG00000171606 | **536** | ENSG00000185340 | **586** | ENSG00000221826 |
| **437** | ENSG00000164761 | **487** | ENSG00000171791 | **537** | ENSG00000185527 | **587** | ENSG00000221926 |
| **438** | ENSG00000164823 | **488** | ENSG00000171819 | **538** | ENSG00000185745 | **588** | ENSG00000223932 |
| **439** | ENSG00000165030 | **489** | ENSG00000171867 | **539** | ENSG00000185823 | **589** | ENSG00000230293 |
| **440** | ENSG00000165059 | **490** | ENSG00000171951 | **540** | ENSG00000186130 | **590** | ENSG00000232159 |
| **441** | ENSG00000165060 | **491** | ENSG00000171960 | **541** | ENSG00000186318 | **591** | ENSG00000232957 |
| **442** | ENSG00000165457 | **492** | ENSG00000172543 | **542** | ENSG00000186470 | **592** | ENSG00000232995 |
| **443** | ENSG00000165583 | **493** | ENSG00000172757 | **543** | ENSG00000186564 | **593** | ENSG00000234487 |
| **444** | ENSG00000165629 | **494** | ENSG00000172818 | **544** | ENSG00000186792 | **594** | ENSG00000235155 |
| **445** | ENSG00000165879 | **495** | ENSG00000173207 | **545** | ENSG00000186810 | **595** | ENSG00000239697 |
| **446** | ENSG00000166147 | **496** | ENSG00000173221 | **546** | ENSG00000187048 | **596** | ENSG00000241644 |
| **447** | ENSG00000166226 | **497** | ENSG00000173660 | **547** | ENSG00000187091 | **597** | ENSG00000241837 |
| **448** | ENSG00000166266 | **498** | ENSG00000173812 | **548** | ENSG00000187446 | **598** | ENSG00000242110 |
| **449** | ENSG00000166444 | **499** | ENSG00000173992 | **549** | ENSG00000188603 | **599** | ENSG00000243477 |
| **450** | ENSG00000166685 | **500** | ENSG00000174307 | **550** | ENSG00000188778 | **600** | ENSG00000243480 |

| **601** | ENSG00000253729 |
| --- | --- |
| **602** | ENSG00000254685 |
| **603** | ENSG00000256060 |
| **604** | ENSG00000256269 |
| **605** | ENSG00000257923 |
| **606** | ENSG00000261371 |
| **607** | ENSG00000263238 |
| **608** | ENSG00000265972 |
| **609** | ENSG00000266412 |
| **610** | ENSG00000266964 |
| **611** | ENSG00000267680 |
| **612** | ENSG00000268500 |
| **613** | ENSG00000270641 |
| **614** | ENSG00000271858 |
| **615** | ENSG00000273564 |
| **616** | ENSG00000274839 |
| **617** | ENSG00000275214 |
| **618** | ENSG00000275365 |
| **619** | ENSG00000275379 |
| **620** | ENSG00000275903 |
| **621** | ENSG00000276180 |
| **622** | ENSG00000276340 |
| **623** | ENSG00000276463 |
| **624** | ENSG00000276701 |
| **625** | ENSG00000276787 |
| **626** | ENSG00000277059 |
| **627** | ENSG00000277334 |
| **628** | ENSG00000277365 |
| **629** | ENSG00000277429 |
| **630** | ENSG00000277804 |
| **631** | ENSG00000278644 |
| **632** | ENSG00000280618 |
| **633** | ENSG00000281320 |
| **634** | ENSG00000282013 |
| **635** | ENSG00000282103 |
| **636** | ENSG00000282230 |
| **637** | ENSG00000282626 |
| **638** | ENSG00000282873 |
| **639** | ENSG00000282928 |
| **640** | ENSG00000282956 |
| **641** | ENSG00000284501 |
| **642** | ENSG00000284916 |
| **643** | ENSG00000285007 |
| **644** | ENSG00000285022 |
| **645** | ENSG00000285193 |
| **646** | ENSG00000285246 |
| **647** | ENSG00000285435 |
| **648** | ENSG00000285456 |
| **649** | ENSG00000287542 |
| **650** | ENSG00000288722 |
